# Unveiling optical soliton solutions and bifurcation analysis in the space–time fractional Fokas–Lenells equation via SSE approach

**DOI:** 10.1038/s41598-024-52308-9

**Published:** 2024-01-23

**Authors:** Ahmed Refaie Ali, Md. Nur Alam, Mst. Wahida Parven

**Affiliations:** 1 Department of Mathematics and Computer Science, Faculty of Science, Menoufia University, Shebin El Kom 32511, Menoufia, Egypt; 2 Department of Mathematics, Pabna University of Science and Technology, Pabna, 6600 Bangladesh

**Keywords:** Nonlinear optics, Solitons, Applied mathematics, Computational science, Applied optics

## Abstract

The space–time fractional Fokas–Lenells (STFFL) equation serves as a fundamental mathematical model employed in telecommunications and transmission technology, elucidating the intricate dynamics of nonlinear pulse propagation in optical fibers. This study employs the Sardar sub-equation (SSE) approach within the STFFL equation framework to explore uncharted territories, uncovering a myriad of optical soliton solutions (OSSs) and conducting a thorough analysis of their bifurcations. The discovered OSSs encompass a diverse array, including bright-dark, periodic, multiple bright-dark solitons, and various other types, forming a captivating spectrum. These solutions reveal an intricate interplay among bright-dark solitons, complex periodic sequences, rhythmic breathers, coexistence of multiple bright-dark solitons, alongside intriguing phenomena like kinks, anti-kinks, and dark-bell solitons. This exploration, built upon meticulous literature review, unveils previously undiscovered wave patterns within the dynamic framework of the STFFL equation, significantly expanding the theoretical understanding and paving the way for innovative applications. Utilizing 2D, contour, and 3D diagrams, we illustrate the influence of fractional and temporal parameters on these solutions. Furthermore, comprehensive 2D, 3D, contour, and bifurcation analysis diagrams scrutinize the nonlinear effects inherent in the STFFL equation. Employing a Hamiltonian function (HF) enables detailed phase-plane dynamics analysis, complemented by simulations conducted using Python and MAPLE software. The practical implications of the discovered OSS solutions extend to real-world physical events, underlining the efficacy and applicability of the SSE scheme in solving time–space nonlinear fractional differential equations (TSNLFDEs). Hence, it is crucial to acknowledge the SSE technique as a direct, efficient, and reliable numerical tool, illuminating precise outcomes in nonlinear comparisons.

## Introduction

Soliton theory constitutes a significant field within nonlinear dynamics, physics, and applied mathematics, impacting various domains such as the telecommunications industry, nonlinear optics, plasma physics, and solid-state physics. Solitons characterize the particle-like properties of nonlinear pulses, and optical solitons, in particular, have garnered attention for their application in transmission technology through diverse waveguide structures. The study of nonlinear optics in the physical sciences and the investigation of optical solitons in communications theory hold great relevance.

Several models have been utilized to develop Optical Solitons (OSs) for communication purposes. The Nonlinear Schrödinger’s equation (NLSE) and its variant forms have been extensively employed in dispersive mediums across various scopes of applied mathematics and physics^1–5^. Solitons emerge due to a delicate equilibrium between group velocity dispersion (GVD) and nonlinearity within the medium. If the GVD value is small, this equilibrium can be at risk. Thus, investigating the balance between these two properties, particularly their dispersive effects, is crucial.

The Fokas–Lenells (FL) equation^6,7^, a prominent model akin to NLSE, finds extensive applications in fiber-optics communication. Its significance in research drives the exploration of diverse forms of OSs. Multiple methodologies have been established to generate OS solutions for such models, including the modified (G′/G)-expansion method^8–10^, the unified method^11,12^, the modified double Laplace transform scheme^13^, the adapted exponential task scheme^14^, the symbolic computational approach^15^, generalized Riccati equation mapping^16^, mapping method^17^, the φ6-model expansion approach^18^, sine–Gordon expansion method^19^, the improved Kudryashov’s approach^20^, Lie symmetry analysis^21^, the New generalized auxiliary equation method^22^, binary Darboux transformations^23^, among many others.

This study aims to explore uncharted Optical Soliton Solutions (OSSs) of the STFFL equation through the local M-derivative and its implications using the SSE^24^, elucidating the dynamic characteristics of the FL model. We consider the STFFL equation via the local M-derivative and its properties as follows^25^:

1.1 $$ \begin{aligned} & iD_{M,t}^{{\alpha ;\delta_{1} }} \psi + a_{1} D_{M,x}^{{2\alpha ;\delta_{1} }} \psi + a_{2} D_{M,t}^{{\alpha ;\delta_{1} }} D_{M,x}^{{\alpha ;\delta_{1} }} \psi + \left| \psi \right|^{2} (\beta \psi + ia_{5} D_{M,x}^{{\alpha ;\delta_{1} }} \psi ) \\ & \quad - ia_{3} D_{M,x}^{{\alpha ;\delta_{1} }} \psi - ia_{4} D_{M,x}^{{\alpha ;\delta_{1} }} (\left| \psi \right|^{2n} \psi ) - i\gamma \psi D_{M,x}^{{\alpha ;\delta_{1} }} (\left| \psi \right|^{2n} ) = 0,\,\,\,\,\,\,\,\,0 < \alpha \le 1,\,n > 0, \\ \end{aligned} $$

where $a_{1}$ is the STD coefficient, $a_{2}$ is GVD coefficient, $a_{4}$ is the self-steepening coefficient,$a_{3}$ is inter-modal dispersion (IMD) coefficient, $\gamma$ is nonlinear dispersion (ND) coefficient, $i = \sqrt { - 1} ,$ $\psi = \psi (x,t)$ is a wave function. The first term of the STFFL equation dictates the fractional temporal evolution of the pulse. When specific condition is met, the STFFL equation reverts to the original FL equation. Within nonlinear optics, the STFFL equation governs the propagation of nonlinear light pulses in monomode optical fibers, incorporating supposed non-linear higher-order properties for a comprehensive explanation.

Numerous researchers^26–59^, as evidenced by a substantial body of recent work, have devoted their efforts to investigating optical solitons concerning space–time fractional phenomena and traveling waves within the domain of telecommunications and transmission technology^26–59^. This reviewed literature spans a wide spectrum of research across various scientific disciplines.

A collection of recent studies has significantly advanced our understanding across physics, mathematics, and engineering disciplines. For instance, Refaie Ali et al.^26^ explored Cherenkov Free-Electron Laser (FEL) reactions in plasma-filled cylindrical waveguides within fractional D-dimensional space, deepening our insights into wave-plasma interactions. Furthermore, studies by Islam et al.^27^, Refaie Ali et al.^28^, Yang et al.^29^, Abdel-Aty et al.^30^, Osman et al.^31^, and Hassan et al.^32^ contributed extensive findings, enhancing our understanding of optical solitons, nonlinear wave phenomena in magnetic systems, electromagnetic wave propagation in plasma environments, and explicit solutions for fractional nonlinear space–time models. These studies, alongside others^26–42^, collectively propel advancements in telecommunications and transmission technology, offering transformative insights into efficient data transmission and introducing novel perspectives on wave behavior within practical applications.

Additionally, a diverse array of research spans various scientific domains. Abdel-Aty et al.^33^ delved into nonlinear multiphoton Jaynes-Cummings model entanglement, while Shapaan^34^ explored semiconductor oxide glasses' conductivity and thermal properties. Other studies by Jayamurugan et al.^35^, Mohamed and Hadia^36^, Thota^37^, Lorenz et al.^38^, Dinesh and Murugesan^39^, Uthayakumar and Gowrisankar^40^, Alam and Refaie^41^, and Khan et al.^42^ cover a wide spectrum of topics, including materials science, mathematical problem-solving tools, fractal aesthetics in architecture, antenna design, image processing methods, fluid dynamics, and soliton dynamics in mathematical physics. This collective body of work significantly enriches scientific knowledge and informs research across various scientific domains. Recent research related to our study has explored diverse phenomena across various equations in mathematical physics. Justin et al.^43^ investigated optical solitons and instability in the Sasa-Satsuma model, shedding light on optical system behaviors. Other studies, such as Bashar et al.^44^ focused on constructing solutions for the (2 + 1)-dimensional Heisenberg ferromagnetic spin chain equation, revealing insights into magnetic system characteristics. Shahen et al.^45^ delved into wave dynamics in the conformable time-fractional modified Kawahara equation, while Mamun et al.^46^ presented precise solutions to 3D fractional WBBM equations. Foyjonnesa, Shahen, and Rahman et al.^47,48^ explored solitary wave structures in the unidirectional DGH equation and computational solutions in an electrical transmission line model. They're joined by Mamun, Shahen et al.^49,50^, who studied wave behaviors in other fractional equations. Additionally, Ghanbari and Gómez‐Aguilar^51,52^ examined optical soliton dynamics in different nonlinear equations, contributing to a deeper understanding of these phenomena across various contexts in mathematical physics. Numerous studies have contributed to various mathematical and physical domains. Ghanbari and Băleanu^53^ explored new optical solutions for the fractional Gerdjikov-Ivanov equation with conformable derivatives. Khater and Ghanbari^54^ focused on solitary wave solutions and gas diffusion characterization in a homogeneous medium. Ghanbari's^55^ studied the abundant soliton solutions for the Hirota–Maccari equation using the generalized exponential rational function method. Ghanbari and Kuo^56^ delved into new exact wave solutions for variable-coefficient equations. Additionally, Alam et al.^57^ explored the dynamics of the Fractional Kraenkel-Manna-Merle System in ferromagnetic materials. Alam et al.^58^ conducted bifurcation and solitary wave analyses of the nonlinear fractional soliton neuron model. Lastly, Alam and Islam^59^ discussed the agreement between novel exact and numerical solutions of nonlinear models.

The manuscript represents a meticulous exploration of the SSE method as an instrumental tool in unraveling the complexities inherent in the STFFL equation. Its primary focus is the comprehensive investigation of a rich spectrum of optical soliton solutions. These solutions encompass an intriguing array, ranging from the duality of bright-dark solitons to the intricate periodicity, the rhythmic breathers, the coexistence of multiple bright-dark solitons, as well as the intriguing phenomena of kinks, anti-kinks, and dark-bell solitons. Through a systematic review and analysis of prior literature, this manuscript unveils hitherto unexplored wave profiles nestled within the intricate folds of the STFFL equation. These revelations promise not only to expand the theoretical foundation but also to offer fertile ground for groundbreaking discoveries and innovative applications. One of the remarkable potentials of this research lies in its capacity to simulate the behavior of extended-wavelength water rollers. This feat is accomplished by deftly incorporating gently nonlinear restorative forces and orchestrating a meticulous distribution of regularities. The implications of such simulations extend far beyond mere emulation, offering profound insights into natural phenomena and facilitating a deeper understanding of wave dynamics across various mediums. Furthermore, the practical applications stemming from this research are vast and impactful. It transcends the realm of theoretical studies, finding relevance and practical utility in critical domains such as optical fiber communication, where the accurate modeling of signal transmission dynamics is paramount. Additionally, its implications ripple across telecommunications, transmission technology, and the nuanced study of nonlinear pulse propagation within optical fibers, promising advancements that could revolutionize these fields. In essence, this manuscript not only contributes significantly to the theoretical understanding of optical solitons but also opens new vistas for practical applications across a spectrum of disciplines, thus bearing the potential to shape the future landscape of multiple scientific and technological domains.

This paper's uniqueness lies in its captivating use of the SSE method, perfectly complementing my insights. Furthermore, the graphical representation of diverse wave number norms and their impact on our configurations enriches our findings substantially. These results hold profound significance, particularly in comprehending ultrashort light pulses within optical fibers.

Our research model carries immense weight across several vital domains: optical fiber communication, signal transmission, telecommunications, transmission technology, and the nonlinear propagation of pulses within optical fibers. Optical soliton phenomena are the cornerstone of the telecommunications industry, and its sustainability hinges on the remarkable soliton transmission technology. Mastering the propagation of optical solitons remains a linchpin for the telecommunications sector, where soliton transmission technology continues to be indispensable for its operations and advancements. This technology constitutes the elemental framework of modern telecommunications, relying on the principles of optical satellite propagation to ensure efficient data transmission and seamless communication.

## Local M-derivative and traveling wave hypothesis

### Local M-derivative

The authors aimed to underscore the paper's originality by addressing why they chose to solve the mentioned equation using the concept of local M-derivative. The authors opted for the local M-derivative over other options like Conformable, fractional, or Beta derivatives, likely due to its specific suitability, applicability, or advantageous characteristics within the context of their study. These derivatives might not have offered the same level of precision, relevance, or effectiveness for addressing the equation or problem outlined in the paper. The local M-derivative offers several advantages:

- Localized Sensitivity: It captures localized behavior within a function, emphasizing changes in a specific region rather than across the entire domain. This localized sensitivity can be crucial in various real-world applications.
- Flexibility: It provides flexibility in describing non-smooth or irregular functions, offering a more nuanced understanding of intricate behaviors within localized segments.
- Adaptability: It can be tailored to various applications, allowing for adjustments based on specific requirements or characteristics of the problem at hand.
- Simplicity: Compared to some other fractional or non-integer order derivatives, the local M-derivative might offer simpler computational procedures or interpretations, facilitating easier implementation and analysis.
- Accuracy: In certain scenarios or for specific types of functions, the local M-derivative might provide more accurate or relevant results compared to other derivative approaches, particularly when focusing on local properties.

These advantages collectively contribute to the appeal and utility of the local M-derivative in certain mathematical or scientific contexts, making it a preferred choice in some studies or analyses.

The application of the local M-derivative enables the transformation of nonlinear fractional partial differential equations into nonlinear fractional ordinary differential equations.

Consider the function $f:[0, \propto ) \to \Re ,$ $t > 0$, $0 < \mu < 1,$ then definition of the local M-derivative of order $\mu$ as follows:

2.1 $$ D_{M}^{\mu ;\delta } \{ f(t)\} = \mathop {\lim }\limits_{\varepsilon \to 0} \frac{{f(tE_{\delta } (\varepsilon t^{ - \mu } )) - f(t)}}{\varepsilon },\quad \forall t > 0, $$

here $E_{\delta } (.)$ is the Mittag–Leffler function with one parameter and $f(t)$ is a p-differentiable function in $(0,p),p > 0,$ and if $\lim_{{t \to 0^{ + } }} D_{M}^{\mu ;\delta }$ exists, subsequently we have

2.2 $$ D_{M}^{\mu ;\delta } \{ f(0)\} = \mathop {\lim }\limits_{{t \to 0^{ + } }} D_{M}^{\mu ;\delta } \{ f(t)\} . $$

The local M-derivative possesses the following properties:

$$ \left\{ \begin{gathered} D_{M}^{\mu ;\delta } \{ f(t)\} = \frac{{t^{1 - \mu } }}{\Gamma (\delta + 1)}\frac{df(t)}{{dt}}, \hfill \\ D_{M}^{\mu ;\delta } \left\{ {\frac{{t^{\mu } \Gamma (\delta + 1)}}{\alpha }} \right\} = 1, \hfill \\ D_{M}^{\mu ;\delta } (f.g)(a) = f^{\prime}(g(a))D_{M}^{\mu ;\delta } \hfill \\ D_{M}^{\mu ;\delta } F\left( {\frac{{\Gamma (\delta + 1)t^{\mu } }}{\mu }} \right) = F^{\prime}\left( {\frac{{\Gamma (\delta + 1)t^{\mu } }}{\mu }} \right)D_{M}^{\mu ;\delta } \left( {\frac{{\Gamma (\delta + 1)t^{\mu } }}{\mu }} \right) = F^{\prime}\left( {\frac{{\Gamma (\delta + 1)t^{\mu } }}{\mu }} \right)\,with\,\eta = \frac{m}{\mu }\Gamma (\delta + 1)t^{\mu } \hfill \\ D_{M}^{\mu ;\delta } F(\eta ) = mF^{\prime}(\eta ). \hfill \\ \end{gathered} \right. $$

### Traveling wave hypothesis

Consider the wave transformation:

2.3 $$ \psi (x,t) = U(\eta )e^{i\Theta } ,\quad \eta = a_{6} \left( {\Gamma (\delta_{1} + 1)\left( {\frac{{x^{\alpha } }}{\alpha } - a_{6} \frac{{t^{\alpha } }}{\alpha }} \right)} \right),\quad \Theta = \left( {\Gamma (\delta_{1} + 1)\left( { - k\frac{{x^{\alpha } }}{\alpha } + \omega \frac{{t^{\alpha } }}{\alpha }} \right)} \right) + \theta . $$

Interchanging equation (2.3) into equation (1.1) and extracting the real part yields

2.4 $$ a_{6}^{2} (a_{1} - a_{2} a_{6} )U^{\prime\prime} + (a_{2} k\omega - \omega - a_{1} k^{2} - a_{3} k)U + (\beta + ka_{5} )U^{3} - ka_{4} U^{1 + 2n} = 0, $$

and from the imaginary part, we get:

2.5 $$ ((a_{6} + a_{3} + 2a_{1} k - a_{2} (a_{6} k + \omega )) - a_{5} U^{2} + (a_{4} + 2na_{4} + 2n\gamma )U^{2n} )U^{\prime} = 0. $$

When n = 1, then the Eqs. (1.1), (2.5) and (2.4) become

2.6 $$ \begin{gathered} iD_{M,t}^{{\alpha ;\delta_{1} }} \psi + a_{1} D_{M,x}^{{2\alpha ;\delta_{1} }} \psi + a_{2} D_{M,t}^{{\alpha ;\delta_{1} }} D_{M,x}^{{\alpha ;\delta_{1} }} \psi + \left| \psi \right|^{2} (\beta \psi + ia_{5} D_{M,x}^{{\alpha ;\delta_{1} }} \psi ) \hfill \\ - ia_{3} D_{M,x}^{{\alpha ;\delta_{1} }} \psi - ia_{4} D_{M,x}^{{\alpha ;\delta_{1} }} (\left| \psi \right|^{2} \psi ) - i\gamma \psi D_{M,x}^{{\alpha ;\delta_{1} }} (\left| \psi \right|^{2} ) = 0,\,\,\,\,\,\,\,\,0 < \alpha \le 1. \hfill \\ \end{gathered} $$

2.7 $$ a_{6}^{2} (a_{1} - a_{2} a_{6} )U^{\prime\prime} + (a_{2} k\omega - \omega - a_{1} k^{2} - a_{3} k)U + (\beta + ka_{5} )U^{3} - ka_{4} U^{3} = 0, $$

2.8 $$ ((a_{6} + a_{3} + 2a_{1} k - a_{2} (a_{6} k + \omega )) - a_{5} U^{2} + (a_{4} + 2na_{4} + 2\gamma )U^{2} )U^{\prime} = 0. $$

Resting $a_{4} + 2na_{4} + 2\gamma = 0$ into Eq. (2.8), we get:

2.9 $$ a_{5} = 3a_{4} + 2\gamma ,\,\,\,a_{6} = \frac{{a_{3} + 2a_{1} k - a_{2} \omega )}}{{a_{2} k - 1}}\,, $$

where $a_{6}$ gives the velocity of solitons.

## Phase plane analysis of the model

Let $S_{1} = a_{6}^{2} (a_{1} - a_{2} a_{6} ),$ $S_{2} = (a_{2} k\omega - \omega - a_{1} k^{2} - a_{3} k)$ and $S_{3} = (\beta + ka_{5} ) - ka_{4}$, then the Eq. (2.7) can be written as:

3.1 $$ S_{1} U^{\prime\prime} + S_{2} U + S_{3} U^{3} = 0. $$

We analyze the phase-plane (PP) dynamics of Eq. (3.1). Therefore, let us consider that $X = U$ and $Y = X^{\prime}$ of the Eq. (3.1) and Eq. (3.1) re-write as a first-order dynamical system (DS) of the form,

3.2 $$ \left\{ \begin{gathered} \frac{dX}{{d\eta }} = Y \hfill \\ \frac{dY}{{d\eta }} = - \frac{{S_{2} }}{{S_{1} }}X - \frac{{S_{3} }}{{S_{1} }}X^{3} , \hfill \\ \end{gathered} \right. $$

which (3.2) expresses the PP through OS solutions of the STFFL equation and $(X(\eta ),\,\,Y(\eta ))$ is the solution of the Eq. (3.1).

The Eq. (3.1) come from the HF in Eq. (3.2) through Hamilton canonical equations (HCEs) $\frac{dX}{{d\eta }} = X^{\prime} = \frac{\partial H}{{\partial Y}}$ and $\frac{dY}{{d\eta }} = Y^{\prime} = - \frac{\partial H}{{\partial X}}$, where $H$ is a $C^{2} -$ function.

3.3 $$ H(X,Y) = \frac{{Y^{2} }}{2} + \frac{{S_{2} }}{{2S_{1} }}X^{2} + \frac{{S_{3} }}{{4S_{1} }}X^{4} . $$

### Theorem 3.1

The values of the Hamiltonian function (HF) are constant along solution curves.

### Proof

We can write $H^{\prime}(X,Y) = H_{X} X^{\prime} + H_{y} Y^{\prime}.$ Applying HCEs, we obtain $H^{\prime}(X,Y) = H_{X} H_{Y} + H_{y} ( - H_{X} ) = 0,$ which suggests $H(X,Y) = cons\tan t( = h).$

Therefore

3.4 $$ H(X,Y) = \frac{{Y^{2} }}{2} + \frac{{S_{2} }}{{2S_{1} }}X^{2} + \frac{{S_{3} }}{{4S_{1} }}X^{4} = h. $$

where h is the constant of integration. The theorem (3.1) is proved.

**Figure 1:**
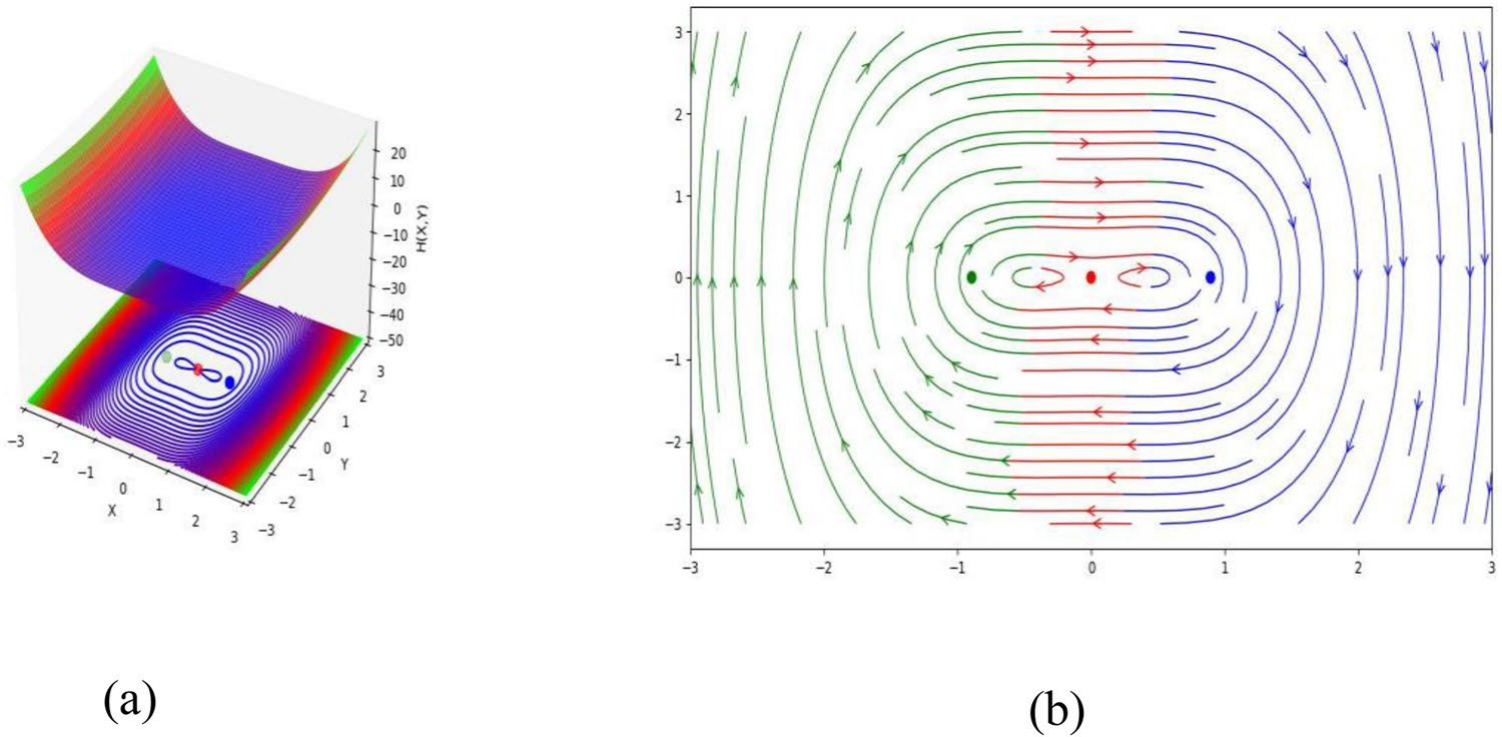
(**a**) HF $H(X,Y)$ of the STFFL equation corresponds to DS (3.2) for the values of $k = 0.50,$ $\omega = 0.30,$ $\beta = 0.25,$ $a_{1} = - 1,$ $a_{2} = - 1.20,$ $a_{3} = - 0.36,$ $a_{4} = 1,$ $a_{5} = 1$ and $a_{6} = 1$; (**b**) PP visualization of DS (3.1) for the values of $k = 0.50,$ $\omega = 0.30,$ $\beta = 0.25,$ $a_{1} = - 1,$ $a_{2} = - 1.20,$ $a_{3} = - 0.36,$ $a_{4} = 1,$ $a_{5} = 1$ and $a_{6} = 1$. Three equilibrium points are $( - 0.8944271910,\,\,0)$, $(0,\,\,0)$ and $(0.8944271910,\,\,0)$.

Three equilibrium points of the DS (3.1) are $(\sqrt {\frac{{ - S_{2} }}{{S_{3} }}} ,0)$, $( - \sqrt {\frac{{ - S_{2} }}{{S_{3} }}} ,0)$ and $(0,0)$. If we set the values of $k = 0.50,$ $\omega = 0.30,$ $\beta = 0.25,$ $a_{1} = - 1,$ $a_{2} = - 1.20,$ $a_{3} = - 0.36,$ $a_{4} = 1,$ $a_{5} = 1$ and $a_{6} = 1$, then the equilibrium points $( - 0.8944271910,\,\,0)$, $(0,\,\,0)$ and $(0.8944271910,\,\,0)$ represent a circle, saddle point and saddle point, respectively (see Fig. 1).

**Figure 2:**
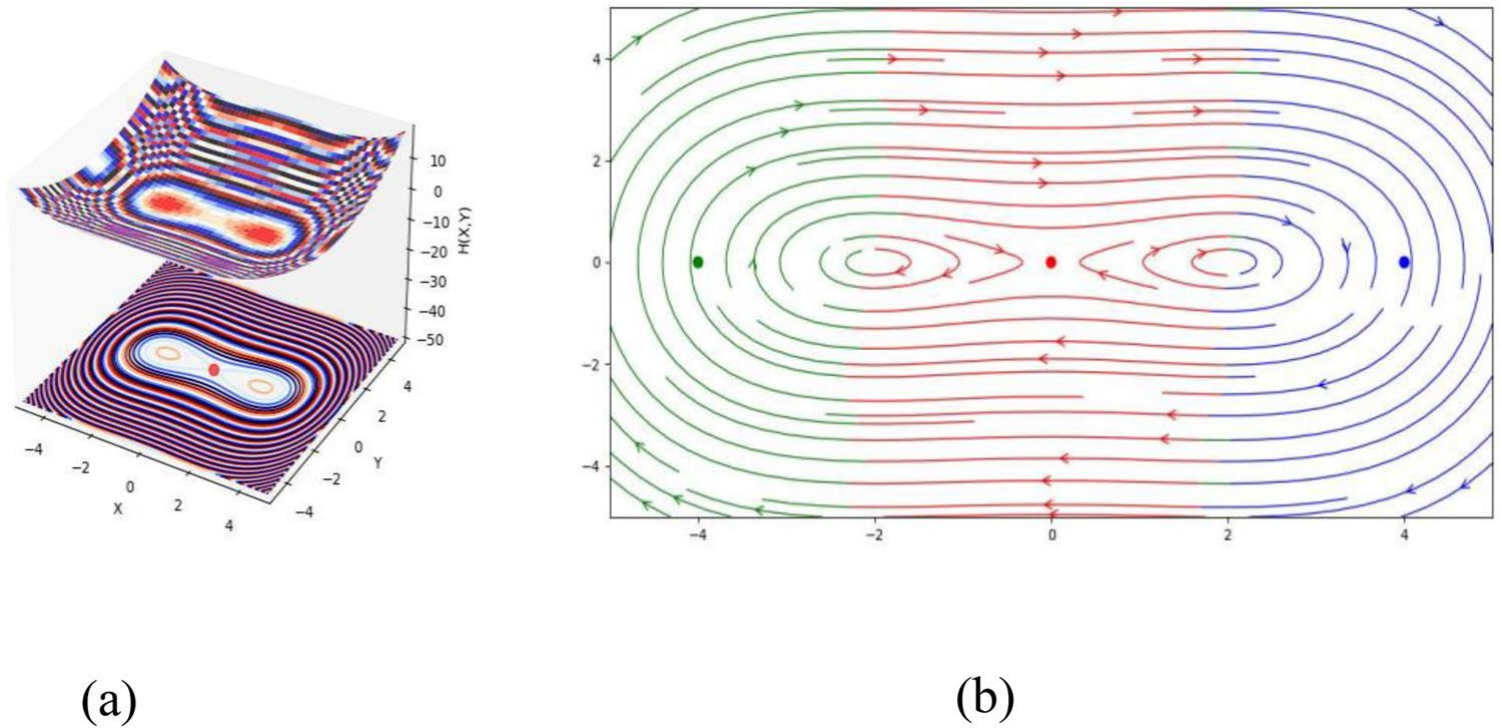
(**a**) HF $H(X,Y)$ of the STFFL equation corresponds to DS (3.2) for the values of $k = 1,$ $\omega = 1,$ $\beta = 0.25,$ $a_{1} = 0,$ $a_{2} = - 4,$ $a_{3} = - 4,$ $a_{4} = 1,$ $a_{5} = 1$ and $a_{6} = 1$; (**b**) PP visualization of the DS (3.1) for the values of $k = 1,$ $\omega = 1,$ $\beta = 0.25,$ $a_{1} = 0,$ $a_{2} = - 4,$ $a_{3} = - 4,$ $a_{4} = 1,$ $a_{5} = 1$ and $a_{6} = 1$. Three equilibrium points are $( - 2,\,\,0)$, $(0,\,\,0)$ and $(2,\,\,0)$.

Three equilibrium points of the DS (3.1) are $(\sqrt {\frac{{ - S_{2} }}{{S_{3} }}} ,0)$, $( - \sqrt {\frac{{ - S_{2} }}{{S_{3} }}} ,0)$ and $(0,0)$. If we set the values of $k = 1,$ $\omega = 1,$ $\beta = 0.25,$ $a_{1} = 0,$ $a_{2} = - 4,$ $a_{3} = - 4,$ $a_{4} = 1,$ $a_{5} = 1$ and $a_{6} = 1$, then the equilibrium points $( - 2,\,\,0)$, $(0,\,\,0)$ and $(2,\,\,0)$ represent a circle, saddle point and saddle point, respectively (see Fig. 2).

**Figure 3:**
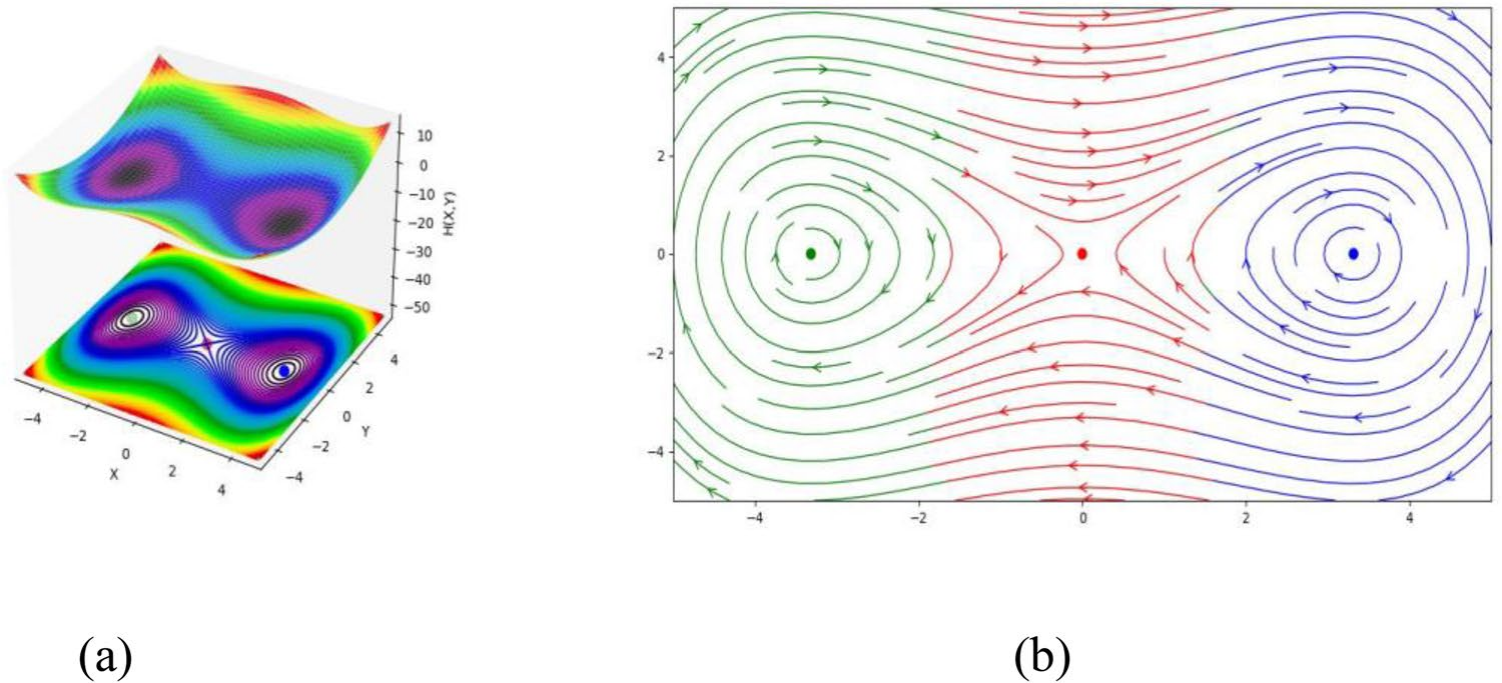
(**a**) HF $H(X,Y)$ of the STFFL equation corresponds to DS (3.2) for the values of $k = 1,$ $\omega = 1,$ $\beta = 1,$ $a_{1} = 10,$ $a_{2} = 1,$ $a_{3} = 1,$ $a_{4} = 1,$ $a_{5} = 1$ and $a_{6} = 1$; (**b**) PP visualization of the DS (3.1) for the values of $k = 1,$ $\omega = 1,$ $\beta = 1,$ $a_{1} = 10,$ $a_{2} = 1,$ $a_{3} = 1,$ $a_{4} = 1,$ $a_{5} = 1$ and $a_{6} = 1$. Three equilibrium points are $( - 3.3166247904,\,\,0)$, $(0,\,\,0)$ and $(3.3166247904,\,\,0)$.

Finally, three equilibrium points of the DS (3.1) are $(\sqrt {\frac{{ - S_{2} }}{{S_{3} }}} ,0)$, $( - \sqrt {\frac{{ - S_{2} }}{{S_{3} }}} ,0)$ and $(0,0)$. If we set the values of $k = 1,$ $\omega = 1,$ $\beta = 1,$ $a_{1} = 10,$ $a_{2} = 1,$ $a_{3} = 1,$ $a_{4} = 1,$ $a_{5} = 1$ and $a_{6} = 1$, then the equilibrium points $( - 3.3166247904,\,\,0)$, $(0,\,\,0)$ and $(3.3166247904,\,\,0)$ represent a circle, saddle point and saddle point, respectively (see Fig. 3). From this discussion, we ascertain that the DS is altered by changing the values of the parameters.

## Analysis of optical soliton solutions via the SSE method

According to the SSE technique, we have:

4.1 $$ U = F_{0} + F_{1} \Phi^{1} $$

From (4.1) and (2.7), then we find:

4.2 $$ a_{1} = 0,\quad a_{2} = \frac{ - \omega }{{\beta \nu^{3} }},\quad \delta = \frac{{ - \omega^{2} }}{{\beta \nu^{3} }},\quad F_{0} = 0,\quad F_{1} = \frac{{ \pm \sqrt { - 2\omega } }}{\beta }. $$

Replacing (4.2) in (4.1), then we obtain:

**Cluster-01** If $a = 0$ and $b > 0$, then

$$ \psi_{1}^{ \pm } (x,t) = \left\{ {\frac{{ \pm \sqrt { - 2\omega } }}{\beta } \times \left( { \pm \sqrt { - \lambda \mu b} \times \frac{2}{{\lambda e^{\sqrt b \eta } + \mu e^{ - \sqrt b \eta } }}} \right)} \right\}e^{i\Theta } . $$

$$ \psi_{2}^{ \pm } (x,t) = \left\{ {\frac{{ \pm \sqrt { - 2\omega } }}{\beta } \times \left( { \pm \sqrt { - \lambda \mu b} \times \frac{2}{{\lambda e^{\sqrt b \eta } - \mu e^{ - \sqrt b \eta } }}} \right)} \right\}e^{i\Theta } . $$

**Cluster-02** If $a = 0$ and $b < 0$, then

$$ \psi_{3}^{ \pm } (x,t) = \left\{ {\frac{{ \pm \sqrt { - 2\omega } }}{\beta } \times \left( { \pm \sqrt { - \lambda \mu b} \times \frac{2}{{\lambda e^{{i\sqrt { - b} \eta }} + \mu e^{{ - i\sqrt { - b} \eta }} }}} \right)} \right\}e^{i\Theta } . $$

$$ \psi_{4}^{ \pm } (x,t) = \left\{ {\frac{{ \pm \sqrt { - 2\omega } }}{\beta } \times \left( { \pm \sqrt { - \lambda \mu b} \times \frac{2}{{\lambda e^{{i\sqrt { - b} \eta }} - \mu e^{{ - i\sqrt { - b} \eta }} }}} \right)} \right\}e^{i\Theta } . $$

**Cluster-03** If $a = \frac{{b^{2} }}{4}$ and $b < 0$, then

$$ \psi_{5}^{ \pm } (x,t) = \left[ {\frac{{ \pm \sqrt { - 2\omega } }}{\beta } \times \left\{ { \pm \sqrt {\frac{ - b}{2}} \times \frac{{\lambda e^{{\sqrt {\frac{ - b}{2}} \eta }} - \mu e^{{ - \sqrt {\frac{ - b}{2}} \eta }} }}{{\lambda e^{{\sqrt {\frac{ - b}{2}} \eta }} + \mu e^{{ - \sqrt {\frac{ - b}{2}} \eta }} }}} \right\}} \right]e^{i\Theta } . $$

$$ \psi_{6}^{ \pm } (x,t) = \left[ {\frac{{ \pm \sqrt { - 2\omega } }}{\beta } \times \left\{ { \pm \sqrt {\frac{ - b}{2}} \times \frac{{\lambda e^{{\sqrt {\frac{ - b}{2}} \eta }} + \mu e^{{ - \sqrt {\frac{ - b}{2}} \eta }} }}{{\lambda e^{{\sqrt {\frac{ - b}{2}} \eta }} - \mu e^{{ - \sqrt {\frac{ - b}{2}} \eta }} }}} \right\}} \right]e^{i\Theta } . $$

$$ \psi_{7}^{ \pm } (x,t) = \left[ {\frac{{ \pm \sqrt { - 2\omega } }}{\beta } \times \left\{ { \pm \sqrt {\frac{ - b}{2}} \times \left\{ {\frac{{\lambda e^{{\sqrt { - 2b} \eta }} - \mu e^{{ - \sqrt { - 2b} \eta }} }}{{\lambda e^{{\sqrt { - 2b} \eta }} + \mu e^{{ - \sqrt { - 2b} \eta }} }} \pm i\sqrt {\lambda \mu } \times \frac{2}{{\lambda e^{{\sqrt { - 2b} \eta }} + \mu e^{{ - \sqrt { - 2b} \eta }} }}} \right\}} \right\}} \right]e^{i\Theta } . $$

$$ \psi_{8}^{ \pm } (x,t) = \left[ {\frac{{ \pm \sqrt { - 2\omega } }}{\beta } \times \left\{ { \pm \sqrt {\frac{ - b}{2}} \times \left\{ {\frac{{\lambda e^{{\sqrt { - 2b} \eta }} + \mu e^{{ - \sqrt { - 2b} \eta }} }}{{\lambda e^{{\sqrt { - 2b} \eta }} - \mu e^{{ - \sqrt { - 2b} \eta }} }} \pm \sqrt {\lambda \mu } \times \frac{2}{{\lambda e^{{\sqrt { - 2b} \eta }} - \mu e^{{ - \sqrt { - 2b} \eta }} }}} \right\}} \right\}} \right]e^{i\Theta } . $$

$$ \psi_{9}^{ \pm } (x,t) = \left[ {\frac{{ \pm \sqrt { - 2\omega } }}{\beta } \times \left\{ { \pm \sqrt {\frac{ - b}{8}} \times \left\{ {\frac{{\lambda e^{{\sqrt {\frac{ - b}{8}} \eta }} - \mu e^{{ - \sqrt {\frac{ - b}{8}} \eta }} }}{{\lambda e^{{\sqrt {\frac{ - b}{8}} \eta }} + \mu e^{{ - \sqrt {\frac{ - b}{8}} \eta }} }} \pm \sqrt {\lambda \mu } \times \frac{{\lambda e^{{\sqrt {\frac{ - b}{8}} \eta }} + \mu e^{{ - \sqrt {\frac{ - b}{8}} \eta }} }}{{\lambda e^{{\sqrt {\frac{ - b}{8}} \eta }} - \mu e^{{ - \sqrt {\frac{ - b}{8}} \eta }} }}} \right\}} \right\}} \right]e^{i\Theta } . $$

**Cluster-04** If $a = \frac{{b^{2} }}{4}$ and $b > 0$, then

$$ \psi_{10}^{ \pm } (x,t) = \left[ {\frac{{ \pm \sqrt { - 2\omega } }}{\beta } \times \left\{ { \pm \sqrt{\frac{b}{2}}  \times \left( { - i\frac{{\lambda e^{{i\sqrt {\frac{ - b}{2}} \eta }} - \mu e^{{ - i\sqrt {\frac{ - b}{2}} \eta }} }}{{\lambda e^{{i\sqrt {\frac{ - b}{2}} \eta }} + \mu e^{{ - i\sqrt {\frac{ - b}{2}} \eta }} }}} \right)} \right\}} \right]e^{i\Theta } . $$

$$ \psi_{11}^{ \pm } (x,t) = \left[ {\frac{{ \pm \sqrt { - 2\omega } }}{\beta } \times \left\{ { \pm \sqrt{\frac{b}{2}}  \times \left( {i\frac{{\lambda e^{{i\sqrt {\frac{ - b}{2}} \eta }} + \mu e^{{ - i\sqrt {\frac{ - b}{2}} \eta }} }}{{\lambda e^{{i\sqrt {\frac{ - b}{2}} \eta }} - \mu e^{{ - i\sqrt {\frac{ - b}{2}} \eta }} }}} \right)} \right\}} \right]e^{i\Theta } . $$

$$ \psi_{12}^{ \pm } (x,t) = \left[ {\frac{{ \pm \sqrt { - 2\omega } }}{\beta } \times \left\{ { \pm \sqrt{\frac{b}{2}}  \times \left( { - i\frac{{\lambda e^{{i\sqrt {2b} \eta }} - \mu e^{{ - i\sqrt {2b} \eta }} }}{{\lambda e^{{i\sqrt {2b} \eta }} + \mu e^{{ - i\sqrt {2b} \eta }} }} \pm i\sqrt {\lambda \mu } \times \frac{2}{{\lambda e^{{i\sqrt {2b} \eta }} + \mu e^{{ - i\sqrt {2b} \eta }} }}} \right)} \right\}} \right]e^{i\Theta } . $$

$$ \psi_{13}^{ \pm } (x,t) = \left[ {\frac{{ \pm \sqrt { - 2\omega } }}{\beta } \times \left\{ { \pm \sqrt{\frac{b}{2}}  \times \left( { - i\frac{{\lambda e^{{i\sqrt {2b} \eta }} + \mu e^{{ - i\sqrt {2b} \eta }} }}{{\lambda e^{{i\sqrt {2b} \eta }} - \mu e^{{ - i\sqrt {2b} \eta }} }} \pm \sqrt {\lambda \mu } \times \frac{2}{{\lambda e^{{i\sqrt {2b} \eta }} - \mu e^{{ - i\sqrt {2b} \eta }} }}} \right)} \right\}} \right]. $$

$$ \psi_{14}^{ \pm } (x,t) = \left[ {\frac{{ \pm \sqrt { - 2\omega } }}{\beta } \times \left\{ { \pm \sqrt{\frac{b}{8}}  \times \left( { - i\frac{{\lambda e^{{i\sqrt{\frac{b}{8}}  \eta }} - \mu e^{{ - i\sqrt{\frac{b}{8}}  \eta }} }}{{\lambda e^{{i\sqrt{\frac{b}{8}}  \eta }} + \mu e^{{ - i\sqrt{\frac{b}{8}}  \eta }} }} \pm \sqrt {\lambda \mu } \times i\frac{{\lambda e^{{i\sqrt{\frac{b}{8}}  \eta }} + \mu e^{{ - i\sqrt{\frac{b}{8}}  \eta }} }}{{\lambda e^{{i\sqrt{\frac{b}{8}}  \eta }} - \mu e^{{ - i\sqrt{\frac{b}{8}}  \eta }} }}} \right)} \right\}} \right]e^{i\Theta } . $$

## Results and discussion

The SSE approach, utilized in this article rather than the improved tan(φ(η)/2)-expansion and the generalized projective Riccati equation methods, holds a key advantage: it generates a broader array of fresh optical soliton solutions, offering extra free parameters. While the solutions obtained from the other methods represent specific instances within the SSE approach, our method introduces entirely new optical soliton solutions, essential for unraveling the underlying mechanisms of physical phenomena. Beyond their significance in the physical realm, these optical soliton solutions derived from nonlinear evolution equations serve as crucial benchmarks for numerical solvers. They aid in comparing result accuracies and conducting stability analyses. In the methods employing the improved tan(φ(η)/2)-expansion and the generalized projective Riccati equation, computer algebra tools like Maple typically assist in finding useful solutions for the algebraic equations in step 4 of Section "Local M-derivative and traveling wave hypothesis", particularly when the reduced ordinary differential equation (ODE) is of order three or less. However, when the order exceeds three, ensuring a solution to the resulting algebraic equations becomes challenging due to the common issue of having more equations than unknowns. Conversely, the SSE method employed here allows for handling reduced ODEs up to the fourth order, thanks to the incorporation of additional arbitrary constants. This capability surpasses the limitations of the improved tan(φ(η)/2)-expansion and generalized projective Riccati equation methods.

In contrast to the research detailed in^25^, our study marks a significant leap in exploring the STFFL equation. Raza et al.^25^ diligently derived eleven solutions using the improved tan(φ(η)/2)-expansion method and seven solutions using the generalized projective Riccati equation method in their comprehensive investigation. Similarly, our study mirrors their findings but unveils a more enriched and expanded set of solutions, representing a pivotal advancement that significantly broadens the spectrum and diversity of potential solutions. We've gone beyond trigonometric and hyperbolic functions, extending into rational, periodic, and hyperbolic forms. Furthermore, our research delves deeper into the intricacies of the STFFL equation by meticulously analyzing the effects of individual parameters, fractional elements, and nonlinearities characterized by general parametric powers on the obtained results. Additionally, we delve into how variations in the order of derivatives influence the resulting wave profile. These investigations are aimed at achieving a deeper understanding of the nature and behavior exhibited by these solutions. Acknowledging the inherent complexity in these findings, we recognize the pivotal role of visual aids in elucidating the essence and dynamics of these solutions. Therefore, we've utilized graphical illustrations to provide an intuitive and accessible comprehension of the intricate relationships and patterns observed within our research. These visual representations serve as indispensable tools, facilitating a comprehensive grasp of the multifaceted nature of the solutions derived from the STFFL equation, thereby enhancing our understanding of their behavior and characteristics. In this section, we present the graphical performance of OSSs in 3D, contour, and 2D surfaces, demonstrating the effects of fractional and time parameters within the STFFL equation.

### 3D graphical representations

**Figure 4:**
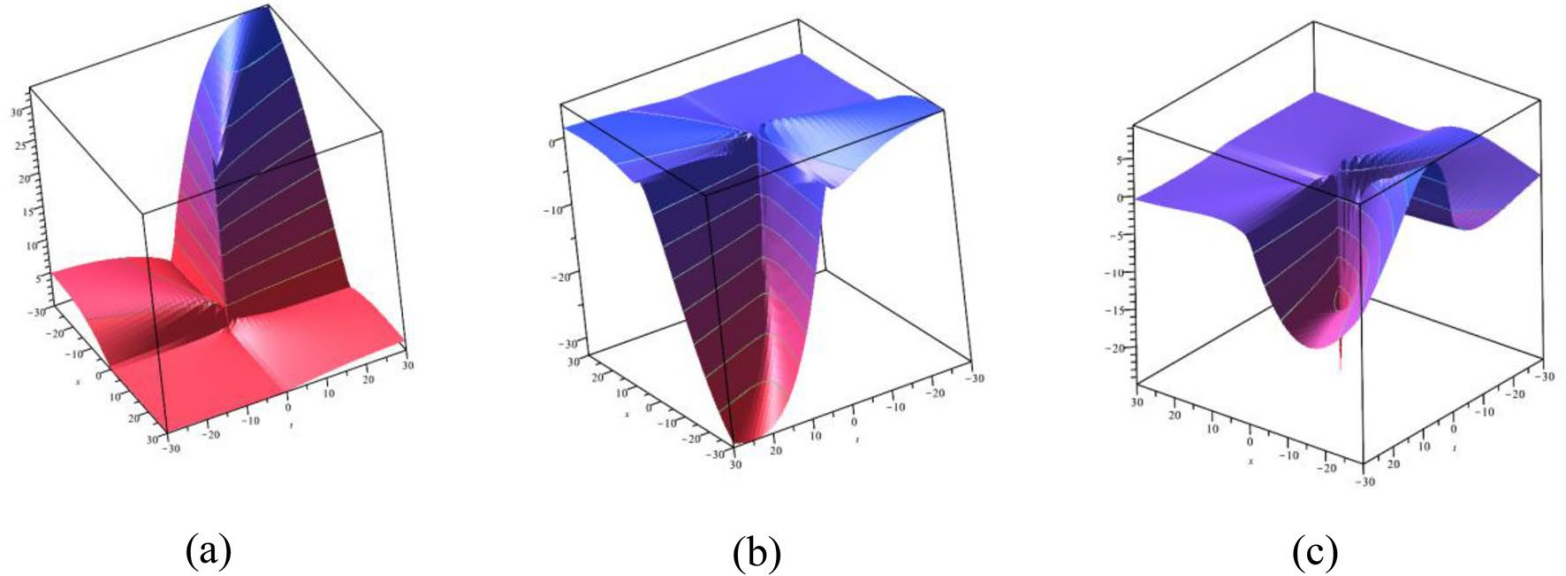
3D [(**a**) Absolute, (**b**) Real, (**c**) Complex] surfaces of $$\psi_{1}^{ \pm } (x,t)$$ with $$\alpha = 
0.10,$$ $$\beta = 0.25,$$ $$\mu = 1,$$ $$\lambda = 2,$$ $$k = 0.50,$$ $$\omega = 0.30,$$ $$\theta = 0.50,$$ $$a_{4} = 1,$$ $$a_{5} = 1,$$ $$a_{6} = 1,$$ $$- 30 \le x \le 30$$, $$- 30 \le t \le 30$$.

**Figure 5:**
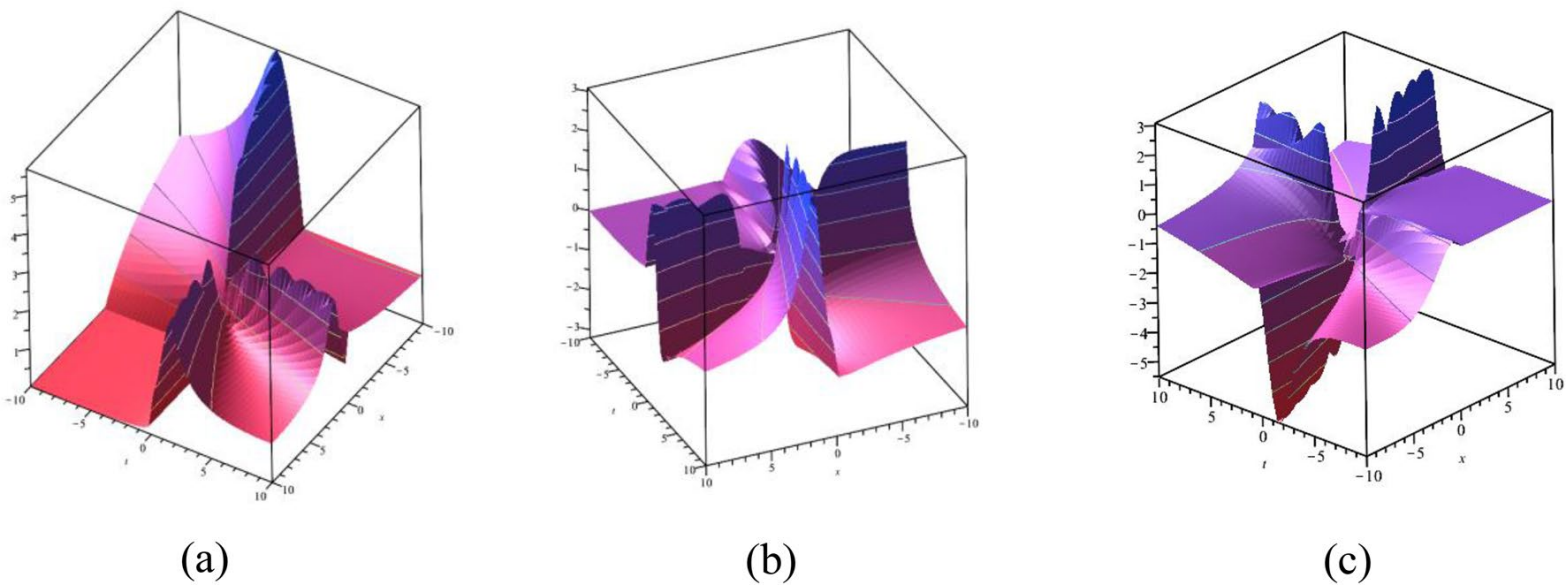
3D [(**a**) Absolute, (**b**) Real, (**c**) Complex] surfaces of $\psi_{3}^{ \pm } (x,t)$ with $\alpha = 0.20,$ $\beta = - 0.50,$ $\mu = 1,$ $\lambda = 2,$ $k = 0.50,$ $\omega = 0.30,$ $\theta = 0.50,$ $a_{4} = 1,$ $a_{5} = 1,$ $a_{6} = 1,$ $- 10 \le x \le 10$, $- 10 \le t \le 10$.

**Figure 6:**
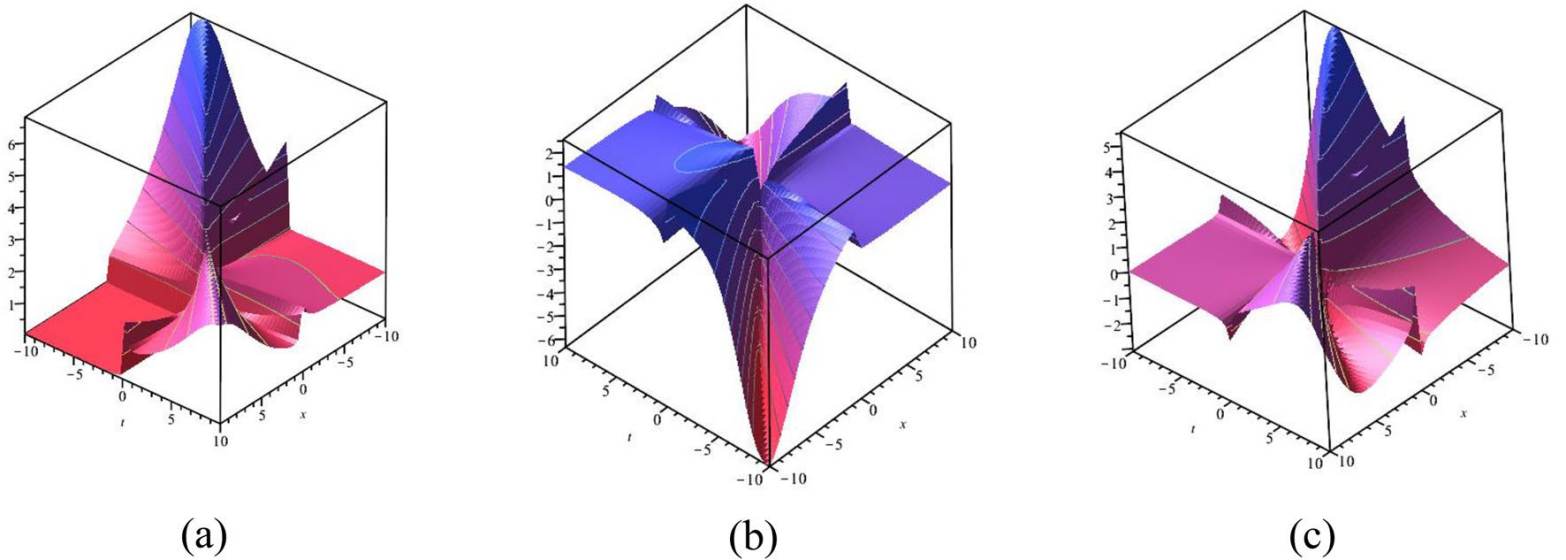
3D [(**a**) Absolute, (**b**) Real, (**c**) Complex] surfaces of $\psi_{4}^{ \pm } (x,t)$ with $\alpha = 0.10,$ $\beta = - 0.50,$ $\mu = 1,$ $\lambda = 2,$ $k = 0.50,$ $\omega = 0.30,$ $\theta = 0.50,$ $a_{4} = 1,$ $a_{5} = 1,$ $a_{6} = 1,$ $- 10 \le x \le 10$, $- 10 \le t \le 10$.

**Figure 7:**
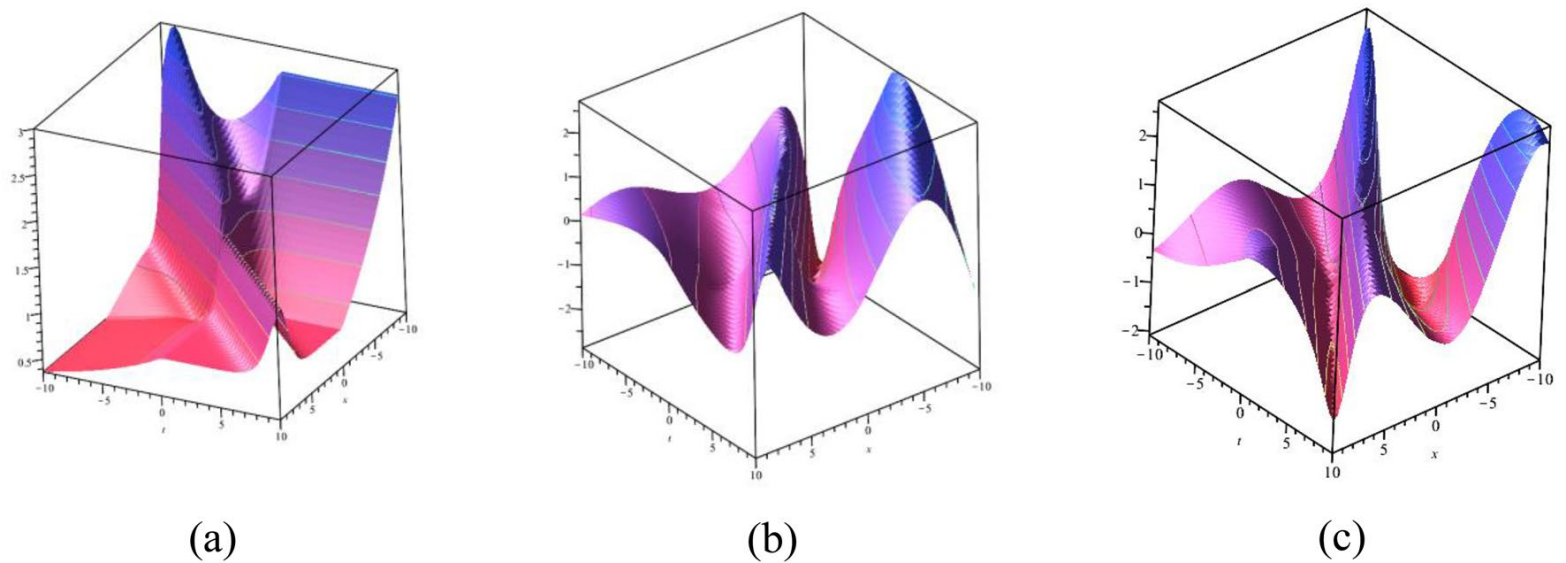
3D [(**a**) Absolute, (**b**) Real, (**c**) Complex] surfaces of $\psi_{7}^{ \pm } (x,t)$ with $\alpha = 0.90,$ $\beta = - 0.50,$ $\mu = 0.10,$ $\lambda = 0.20,$ $k = 0.50,$ $\omega = 0.30,$ $\theta = 0.50,$ $a_{4} = 1,$ $a_{5} = 1,$ $a_{6} = 1,$ $- 10 \le x \le 10$, $- 10 \le t \le 10$.

**Figure 8:**
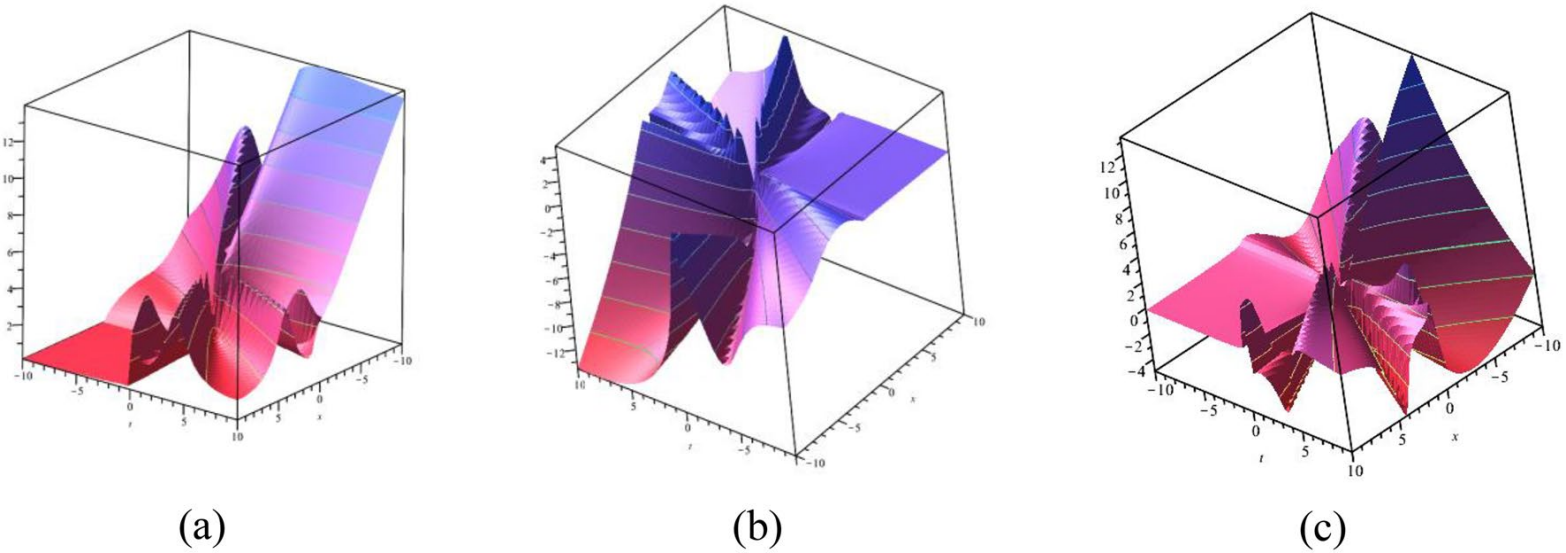
3D [(**a**) Absolute, (**b**) Real, (**c**) Complex] surfaces of $\psi_{12}^{ \pm } (x,t)$ with $\alpha = 0.25,$ $\beta = 0.50,$ $\mu = 1,$ $\lambda = 2,$ $k = 0.50,$ $\omega = 0.30,$ $\theta = 0.50,$ $a_{4} = 1,$ $a_{5} = 1,$ $a_{6} = 1,$ $- 10 \le x \le 10$, $- 10 \le t \le 10$.

In this subsection, we display the graphical performance of OSSs on 3D surfaces. Figure 4a–c illustrate the physical appearance of $\psi_{1}^{ \pm } (x,t)$ in the 3D [(a) Absolute, (b) Real, (c) Complex] surfaces with $\alpha = 0.10,$ $\beta = 0.25,$ $\mu = 1,$ $\lambda = 2,$ $k = 0.50,$ $\omega = 0.30,$ $\theta = 0.50,$ $a_{4} = 1,$ $a_{5} = 1,$ $a_{6} = 1,$ $- 30 \le x \le 30$, $- 30 \le t \le 30$. The solution $\psi_{1}^{ \pm } (x,t)$ provides the bright and dark optical soliton solution. Figure 5a–c presents the physical appearance of $\psi_{3}^{ \pm } (x,t)$ in the 3D [(a) Absolute, (b) Real, (c) Complex] surfaces with $\alpha = 0.20,$ $\beta = - 0.50,$ $\mu = 1,$ $\lambda = 2,$ $k = 0.50,$ $\omega = 0.30,$ $\theta = 0.50,$ $a_{4} = 1,$ $a_{5} = 1,$ $a_{6} = 1,$ $- 10 \le x \le 10$, $- 10 \le t \le 10$. The solution $\psi_{3}^{ \pm } (x,t)$ provides the multiple bright and dark optical soliton solution. Figure 6a–c presents the physical appearance of $\psi_{4}^{ \pm } (x,t)$ in the 3D [(a) Absolute, (b) Real, (c) Complex] surfaces with $\alpha = 0.10,$ $\beta = - 0.50,$ $\mu = 1,$ $\lambda = 2,$ $k = 0.50,$ $\omega = 0.30,$ $\theta = 0.50,$ $a_{4} = 1,$ $a_{5} = 1,$ $a_{6} = 1,$ $- 10 \le x \le 10$, $- 10 \le t \le 10$. The solution $\psi_{4}^{ \pm } (x,t)$ provides the multiple bright and dark optical soliton solution. Figure 7a–c presents the physical appearance of $\psi_{7}^{ \pm } (x,t)$ in the 3D [(a) Absolute, (b) Real, (c) Complex] surfaces with $\alpha = 0.90,$ $\beta = - 0.50,$ $\mu = 0.10,$ $\lambda = 0.20,$ $k = 0.50,$ $\omega = 0.30,$ $\theta = 0.50,$ $a_{4} = 1,$ $a_{5} = 1,$ $a_{6} = 1,$ $- 10 \le x \le 10$, $- 10 \le t \le 10$. The solution $\psi_{7}^{ \pm } (x,t)$ provides the periodic optical soliton solution. Figure 8a–c presents the physical appearance of $\psi_{12}^{ \pm } (x,t)$ in the 3D [(a) Absolute, (b) Real, (c) Complex] surfaces with $\alpha = 0.25,$ $\beta = 0.50,$ $\mu = 1,$ $\lambda = 2,$ $k = 0.50,$ $\omega = 0.30,$ $\theta = 0.50,$ $a_{4} = 1,$ $a_{5} = 1,$ $a_{6} = 1,$ $- 10 \le x \le 10$, $- 10 \le t \le 10$. The solution $\psi_{12}^{ \pm } (x,t)$ provides the multiple bright optical soliton solution.

### Contour graphical representations for different value for time

**Figure 9:**
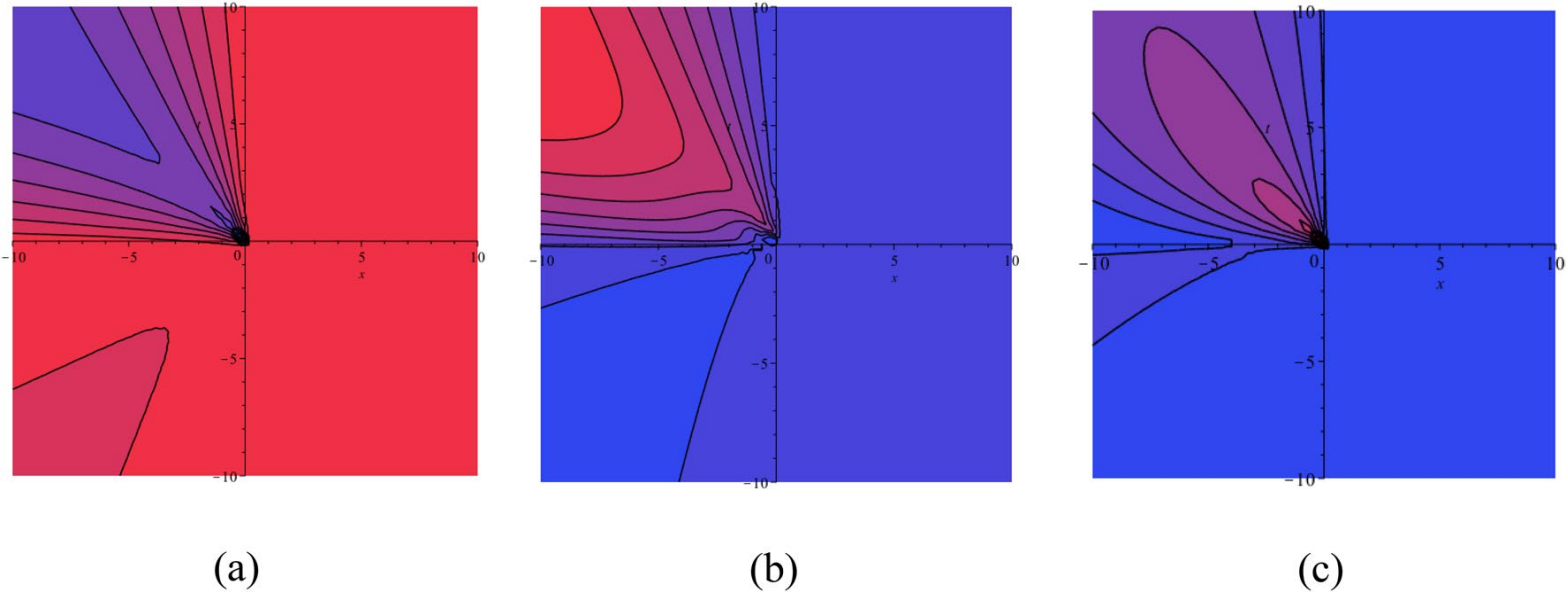
Contour [(**a**) Absolute, (**b**) Real, (**c**) Complex] surfaces of $\psi_{1}^{ \pm } (x,t)$ with $\alpha = 0.10,$ $\beta = 0.25,$ $\mu = 1,$ $\lambda = 2,$ $k = 0.50,$ $\omega = 0.30,$ $\theta = 0.50,$ $a_{4} = 1,$ $a_{5} = 1,$ $a_{6} = 1,$ $- 30 \le x \le 30$, $- 30 \le t \le 30$.

**Figure 10:**
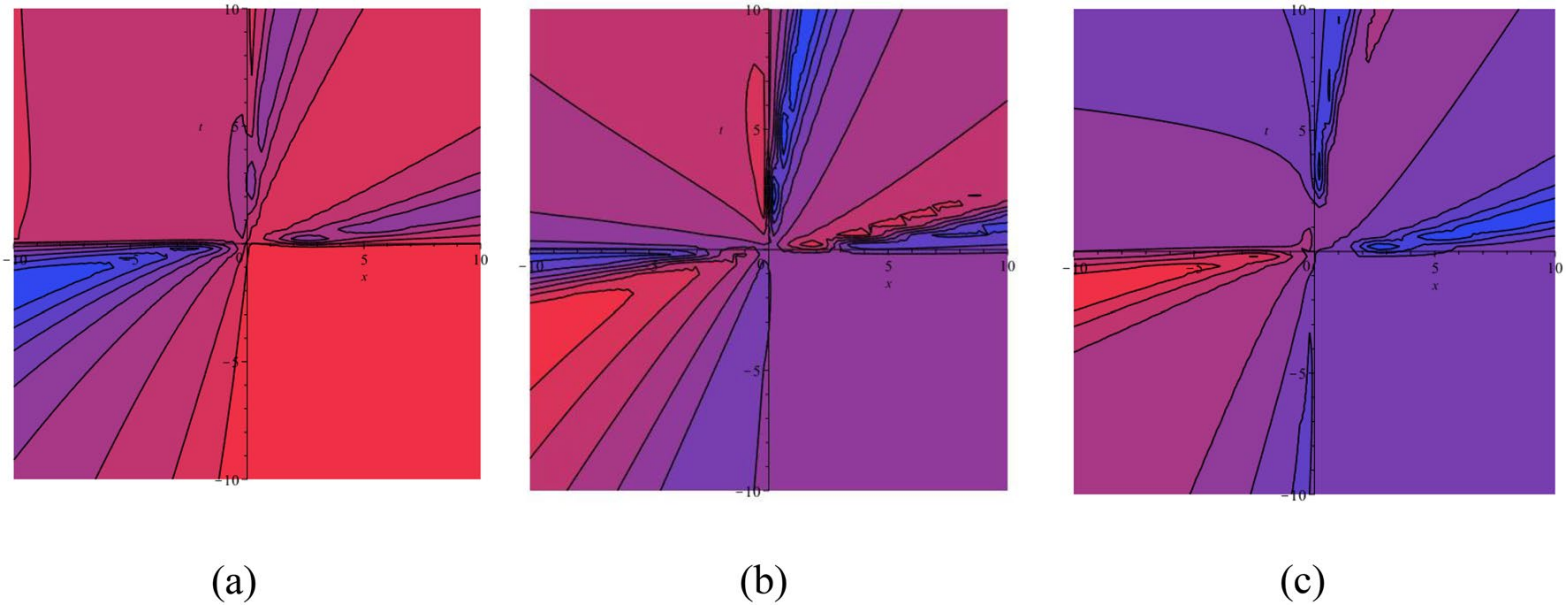
Contour [(**a**) Absolute, (**b**) Real, (**c**) Complex] surfaces of $\psi_{3}^{ \pm } (x,t)$ with $\alpha = 0.20,$ $\beta = - 0.50,$ $\mu = 1,$ $\lambda = 2,$ $k = 0.50,$ $\omega = 0.30,$ $\theta = 0.50,$ $a_{4} = 1,$ $a_{5} = 1,$ $a_{6} = 1,$ $- 10 \le x \le 10$, $- 10 \le t \le 10$.

**Figure 11:**
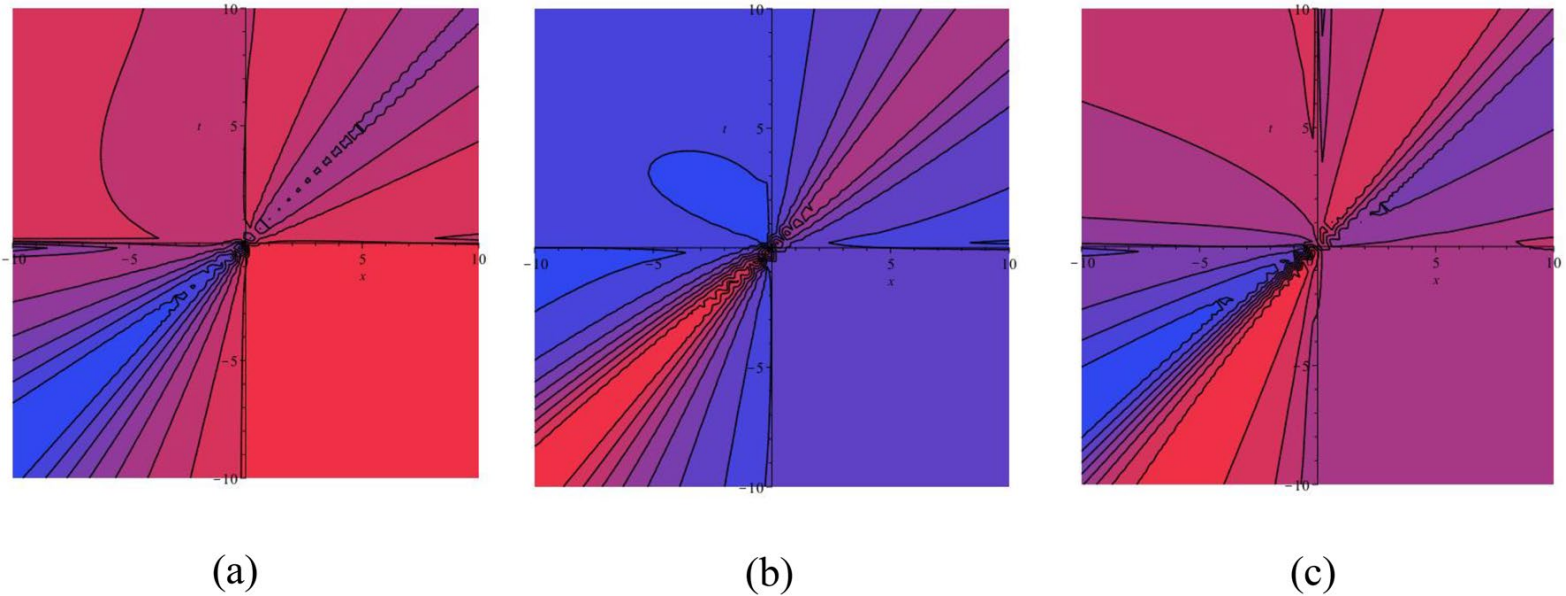
Contour [(**a**) Absolute, (**b**) Real, (**c**) Complex] surfaces of $\psi_{4}^{ \pm } (x,t)$ with $\alpha = 0.10,$ $\beta = - 0.50,$ $\mu = 1,$ $\lambda = 2,$ $k = 0.50,$ $\omega = 0.30,$ $\theta = 0.50,$ $a_{4} = 1,$ $a_{5} = 1,$ $a_{6} = 1,$ $- 10 \le x \le 10$, $- 10 \le t \le 10$.

**Figure 12:**
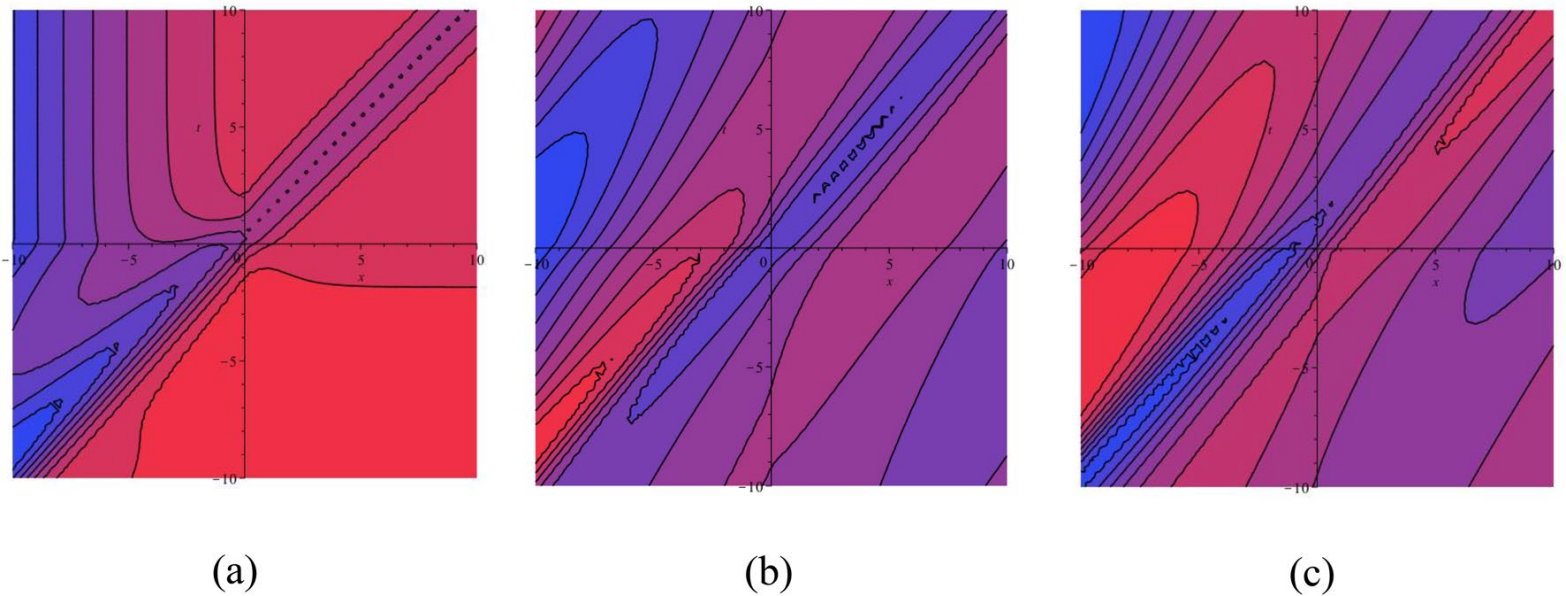
Contour [(**a**) Absolute, (**b**) Real, (**c**) Complex] surfaces of $\psi_{7}^{ \pm } (x,t)$ with $\alpha = 0.90,$ $\beta = - 0.50,$ $\mu = 0.10,$ $\lambda = 0.20,$ $k = 0.50,$ $\omega = 0.30,$ $\theta = 0.50,$ $a_{4} = 1,$ $a_{5} = 1,$ $a_{6} = 1,$ $- 10 \le x \le 10$, $- 10 \le t \le 10$.

**Figure 13:**
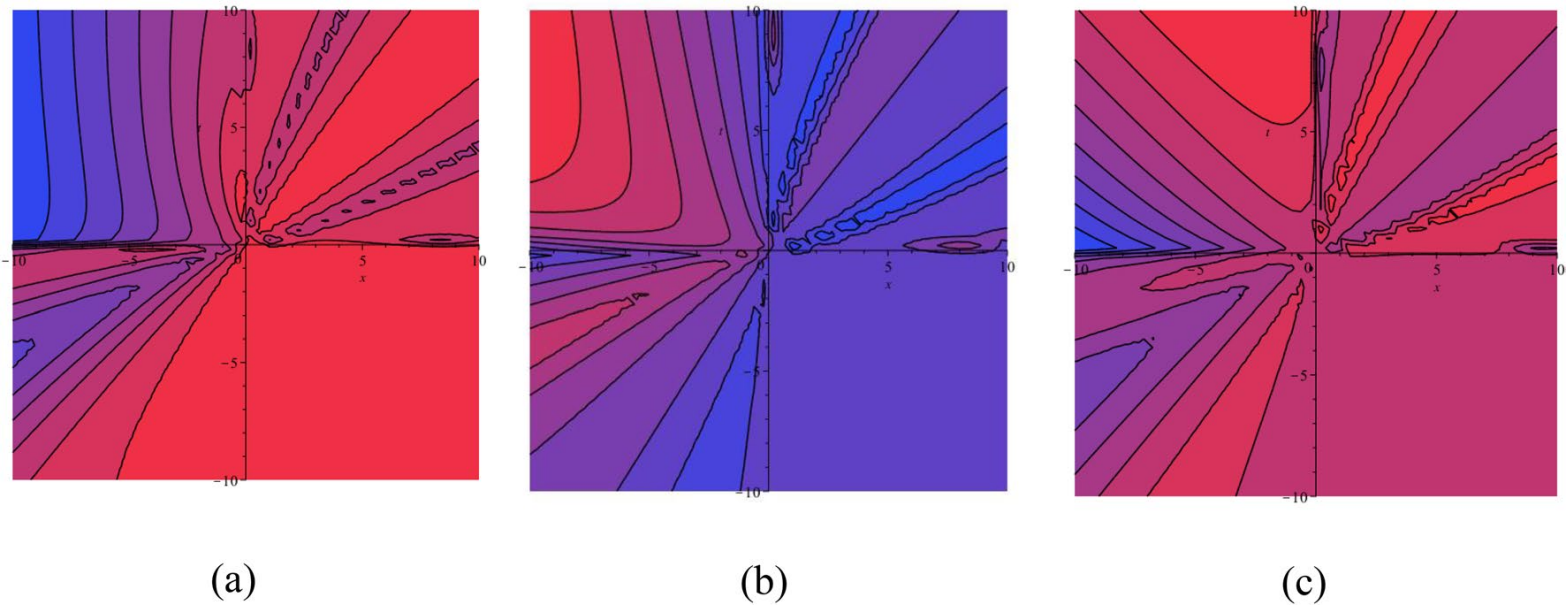
Contour [(**a**) Absolute, (**b**) Real, (**c**) Complex] surfaces of $\psi_{12}^{ \pm } (x,t)$ with $\alpha = 0.25,$ $\beta = 0.50,$ $\mu = 1,$ $\lambda = 2,$ $k = 0.50,$ $\omega = 0.30,$ $\theta = 0.50,$ $a_{4} = 1,$ $a_{5} = 1,$ $a_{6} = 1,$ $- 10 \le x \le 10$, $- 10 \le t \le 10$.

In this subsection gave the graphically performance of OSSs in the contour surfaces. Figure 9a–c presents the physical appearance of $\psi_{1}^{ \pm } (x,t)$ in the contour [(a) Absolute, (b) Real, (c) Complex] surfaces with $\alpha = 0.10,$ $\beta = 0.25,$ $\mu = 1,$ $\lambda = 2,$ $k = 0.50,$ $\omega = 0.30,$ $\theta = 0.50,$ $a_{4} = 1,$ $a_{5} = 1,$ $a_{6} = 1,$ $- 30 \le x \le 30$, $- 30 \le t \le 30$. Figure 10a–c presents the physical appearance of $\psi_{3}^{ \pm } (x,t)$ in the contour [(a) Absolute, (b) Real, (c) Complex] surfaces with $\alpha = 0.20,$ $\beta = - 0.50,$ $\mu = 1,$ $\lambda = 2,$ $k = 0.50,$ $\omega = 0.30,$ $\theta = 0.50,$ $a_{4} = 1,$ $a_{5} = 1,$ $a_{6} = 1,$ $- 10 \le x \le 10$, $- 10 \le t \le 10$. Figure 11a–c presents the physical appearance of $\psi_{4}^{ \pm } (x,t)$ in the contour [(a) Absolute, (b) Real, (c) Complex] surfaces with $\alpha = 0.10,$ $\beta = - 0.50,$ $\mu = 1,$ $\lambda = 2,$ $k = 0.50,$ $\omega = 0.30,$ $\theta = 0.50,$ $a_{4} = 1,$ $a_{5} = 1,$ $a_{6} = 1,$ $- 10 \le x \le 10$, $- 10 \le t \le 10$. Figure 12a–c presents the physical appearance of $\psi_{7}^{ \pm } (x,t)$ in the contour [(a) Absolute, (b) Real, (c) Complex] surfaces with $\alpha = 0.90,$ $\beta = - 0.50,$ $\mu = 0.10,$ $\lambda = 0.20,$ $k = 0.50,$ $\omega = 0.30,$ $\theta = 0.50,$ $a_{4} = 1,$ $a_{5} = 1,$ $a_{6} = 1,$ $- 10 \le x \le 10$, $- 10 \le t \le 10$. Figure 13a–c presents the physical appearance of $\psi_{12}^{ \pm } (x,t)$ in the contour [(a) Absolute, (b) Real, (c) Complex] surfaces with $\alpha = 0.25,$ $\beta = 0.50,$ $\mu = 1,$ $\lambda = 2,$ $k = 0.50,$ $\omega = 0.30,$ $\theta = 0.50,$ $a_{4} = 1,$ $a_{5} = 1,$ $a_{6} = 1,$ $- 10 \le x \le 10$, $- 10 \le t \le 10$.

### 2D graphical representations for different value for time

**Figure 14:**
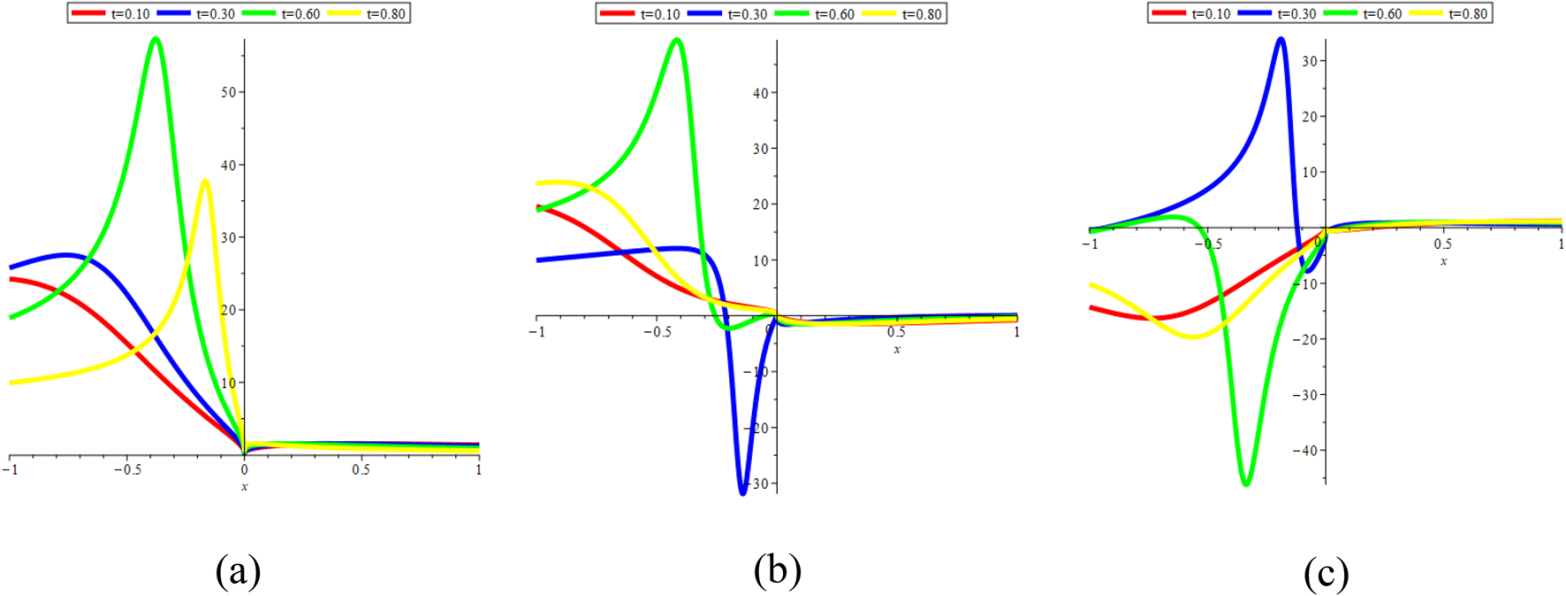
2D [(**a**) Absolute, (**b**) Real, (**c**) Complex] surfaces of $\psi_{1}^{ \pm } (x,t)$ for $t = 0.10,\,\,0.30,\,\,0.60,\,\,0.80$ with $\alpha = 0.20,$ $\beta = 0.25,$ $\mu = 1,$ $\lambda = 2,$ $k = 0.50,$ $\omega = 0.30,$ $\theta = 0.50,$ $a_{4} = 1,$ $a_{5} = 1,$ $a_{6} = 1,$ $- 30 \le x \le 30$.

In this sub-section, provide the effect of time parameters of the STFFL equation. In Fig. 14a–c, we present 2D [(a) Absolute, (b) Real, (c) Complex] surfaces of $\psi_{1}^{ \pm } (x,t)$ with different values of $t = 0.10$ is represent the red line, $t = 0.30$ is represent the blue line, $t = 0.60$ is represent the green and $t = 0.80$ is represent the yellow with $\alpha = 0.20,$ $\beta = 0.25,$ $\mu = 1,$ $\lambda = 2,$ $k = 0.50,$ $\omega = 0.30,$ $\theta = 0.50,$ $a_{4} = 1,$ $a_{5} = 1,$ $a_{6} = 1,$ $- 30 \le x \le 30$.

**Figure 15:**
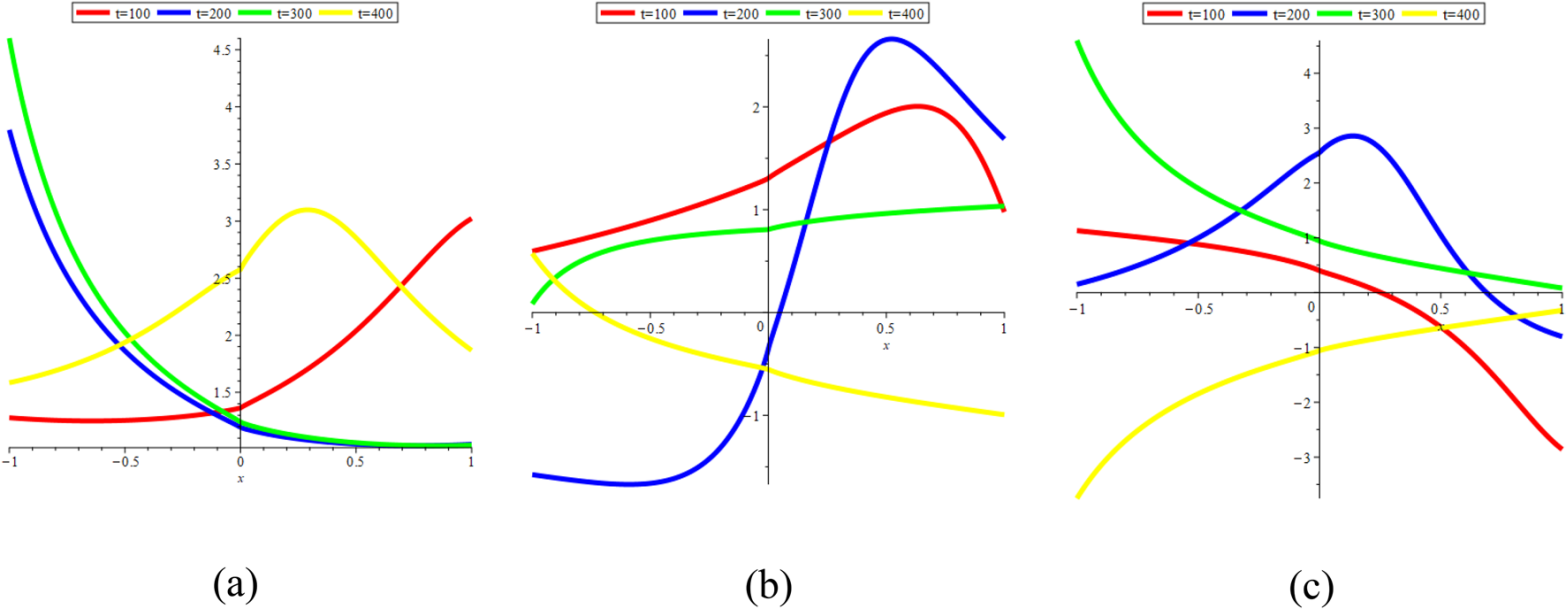
2D [(**a**) Absolute, (**b**) Real, (**c**) Complex] surfaces of $\psi_{3}^{ \pm } (x,t)$ for $t = 100,\,\,200,\,\,300,\,\,400$ with $\alpha = 0.90,$ $\beta = - 0.50,$ $\mu = 1,$ $\lambda = 2,$ $k = 0.50,$ $\omega = 0.30,$ $\theta = 0.50,$ $a_{4} = 1,$ $a_{5} = 1,$ $a_{6} = 1,$ $- 10 \le x \le 10$.

In Fig. 15a–c, we present 2D [(a) Absolute, (b) Real, (c) Complex] surfaces of $\psi_{3}^{ \pm } (x,t)$ with different values of $t = 100$ is represent the red line, $t = 200$ is represent the blue line, $t = 300$ is represent the green and $t = 400$ is represent the yellow with $\alpha = 0.90,$ $\beta = - 0.50,$ $\mu = 1,$ $\lambda = 2,$ $k = 0.50,$ $\omega = 0.30,$ $\theta = 0.50,$ $a_{4} = 1,$ $a_{5} = 1,$ $a_{6} = 1,$ $- 10 \le x \le 10$.

**Figure 16:**
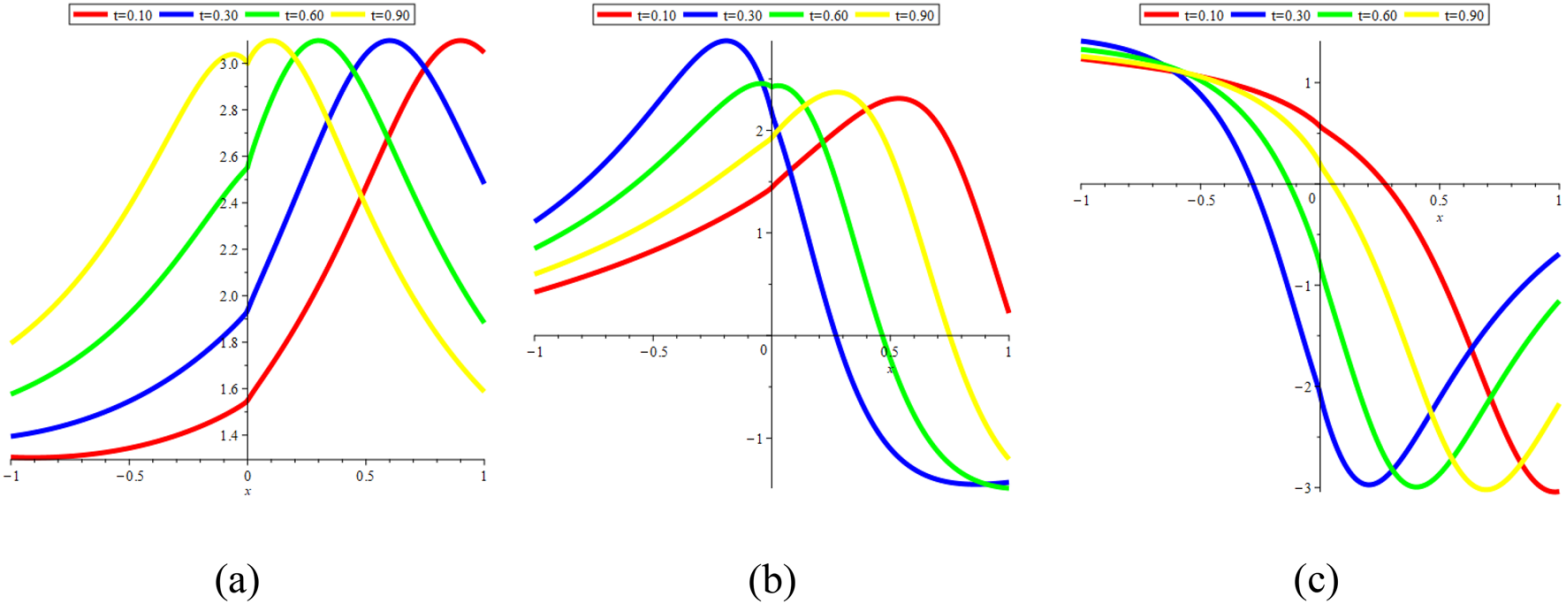
2D [(**a**) Absolute, (**b**) Real, (**c**) Complex] surfaces of $\psi_{4}^{ \pm } (x,t)$ for $t = 10,\,\,30,\,\,60,\,\,80$ with $\alpha = 0.90,$ $\beta = - 0.50,$ $\mu = 1,$ $\lambda = 2,$ $k = 0.50,$ $\omega = 0.30,$ $\theta = 0.50,$ $a_{4} = 1,$ $a_{5} = 1,$ $a_{6} = 1,$ $- 10 \le x \le 10$.

In Fig. 16a–c, we present 2D [(a) Absolute, (b) Real, (c) Complex] surfaces of $\psi_{4}^{ \pm } (x,t)$ with different values of $t = 10$ is represent the red line, $t = 30$ is represent the blue line, $t = 60$ is represent the green and $t = 80$ is represent the yellow with $\alpha = 0.90,$ $\beta = - 0.50,$ $\mu = 1,$ $\lambda = 2,$ $k = 0.50,$ $\omega = 0.30,$ $\theta = 0.50,$ $a_{4} = 1,$ $a_{5} = 1,$ $a_{6} = 1,$ $- 10 \le x \le 10$.

**Figure 17:**
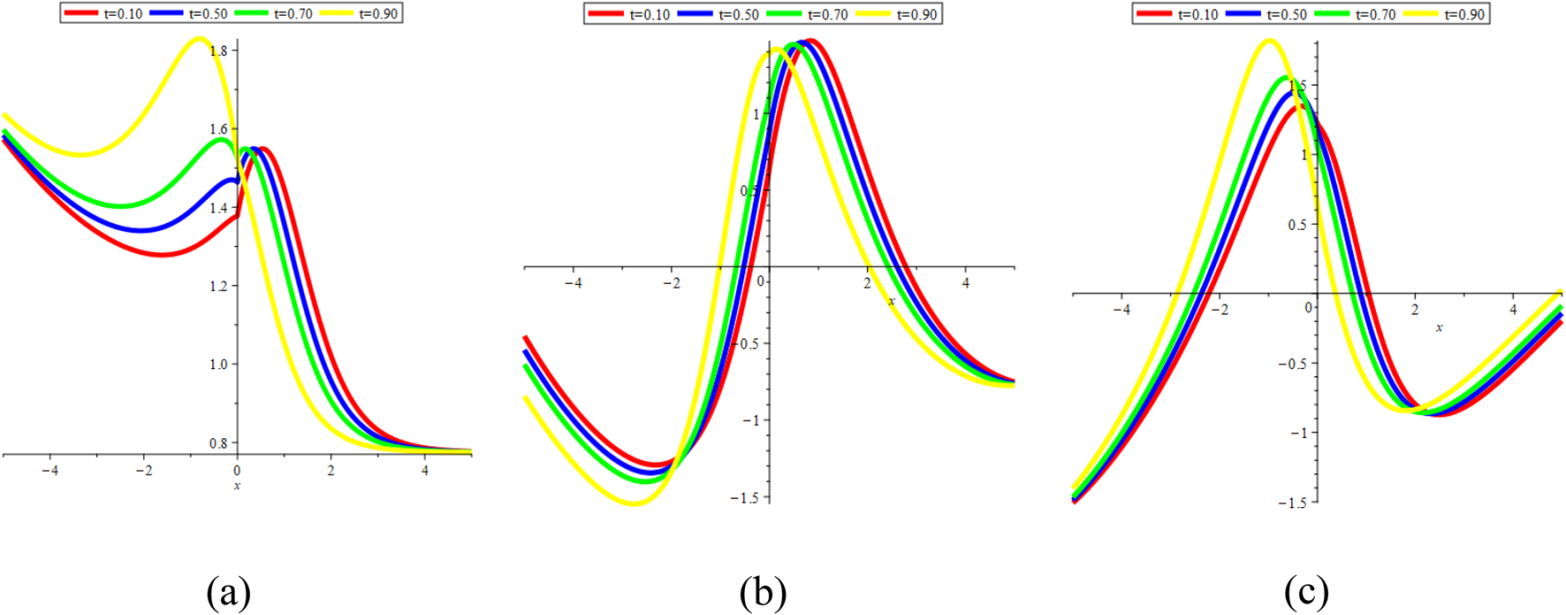
2D [(**a**) Absolute, (**b**) Real, (**c**) Complex] surfaces of $\psi_{7}^{ \pm } (x,t)$ for $t = 0.10,\,\,0.50,\,\,0.70,\,\,0.90$ with $\alpha = 0.20,$ $\beta = - 0.50,$ $\mu = 0.10,$ $\lambda = 0.20,$ $k = 0.50,$ $\omega = 0.30,$ $\theta = 0.50,$ $a_{4} = 1,$ $a_{5} = 1,$ $a_{6} = 1,$ $- 10 \le x \le 10$.

In Fig. 17a–c, we present 2D [(a) Absolute, (b) Real, (c) Complex] surfaces of $\psi_{7}^{ \pm } (x,t)$ with different values of $t = 0.10$ is represent the red line, $t = 0.50$ is represent the blue line, $t = 0.70$ is represent the green and $t = 0.90$ is represent the yellow with $\alpha = 0.20,$ $\beta = - 0.50,$ $\mu = 0.10,$ $\lambda = 0.20,$ $k = 0.50,$ $\omega = 0.30,$ $\theta = 0.50,$ $a_{4} = 1,$ $a_{5} = 1,$ $a_{6} = 1,$ $- 10 \le x \le 10$.

**Figure 18:**
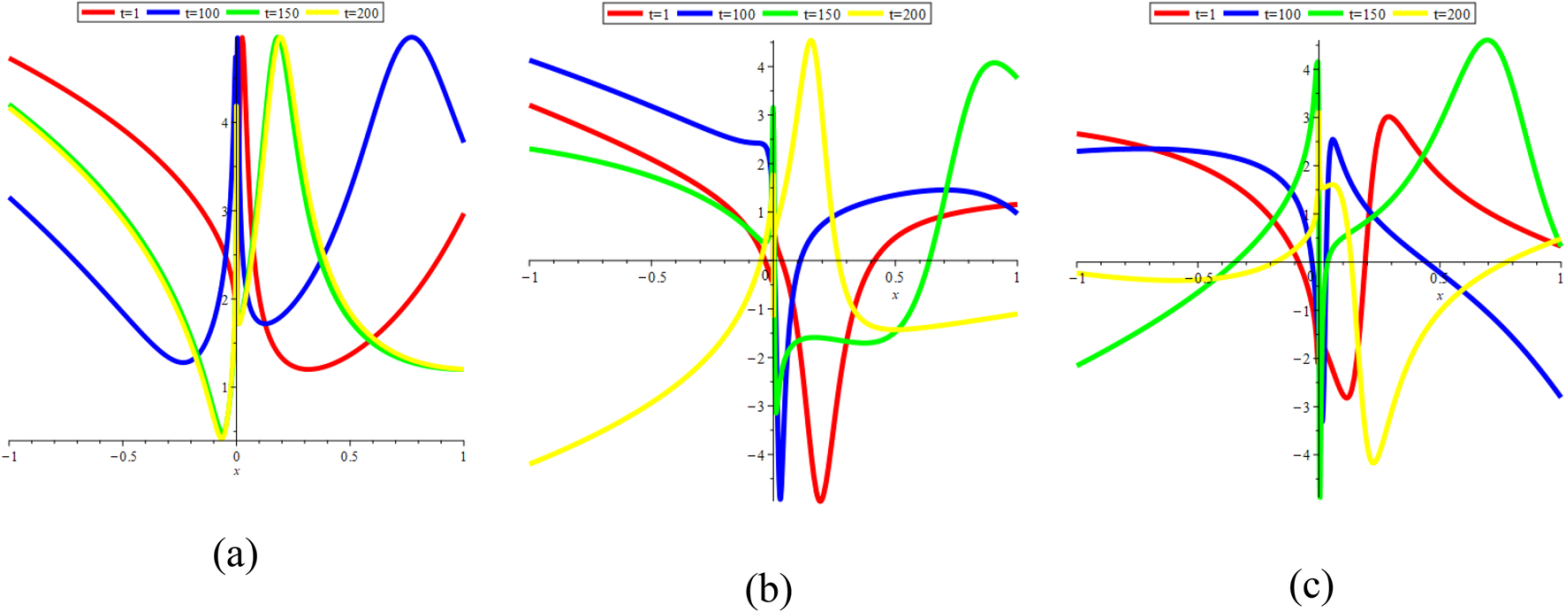
2D [(**a**) Absolute, (**b**) Real, (**c**) Complex] surfaces of $\psi_{12}^{ \pm } (x,t)$ for $t = 1,\,\,100,\,\,150,\,\,200$ with $\alpha = 0.25,$ $\beta = 0.50,$ $\mu = 1,$ $\lambda = 2,$ $k = 0.50,$ $\omega = 0.30,$ $\theta = 0.50,$ $a_{4} = 1,$ $a_{5} = 1,$ $a_{6} = 1,$ $- 10 \le x \le 10$.

In Fig. 18a–c, we present 2D [(a) Absolute, (b) Real, (c) Complex] surfaces of $\psi_{12}^{ \pm } (x,t)$ with different values of $t = 1$ is represent the red line, $t = 100$ is represent the blue line, $t = 150$ is represent the green and $t = 200$ is represent the yellow with $\alpha = 0.25,$ $\beta = 0.50,$ $\mu = 1,$ $\lambda = 2,$ $k = 0.50,$ $\omega = 0.30,$ $\theta = 0.50,$ $a_{4} = 1,$ $a_{5} = 1,$ $a_{6} = 1,$ $- 10 \le x \le 10$.

### 3D graphical representations for different value for fractional order

**Figure 19:**
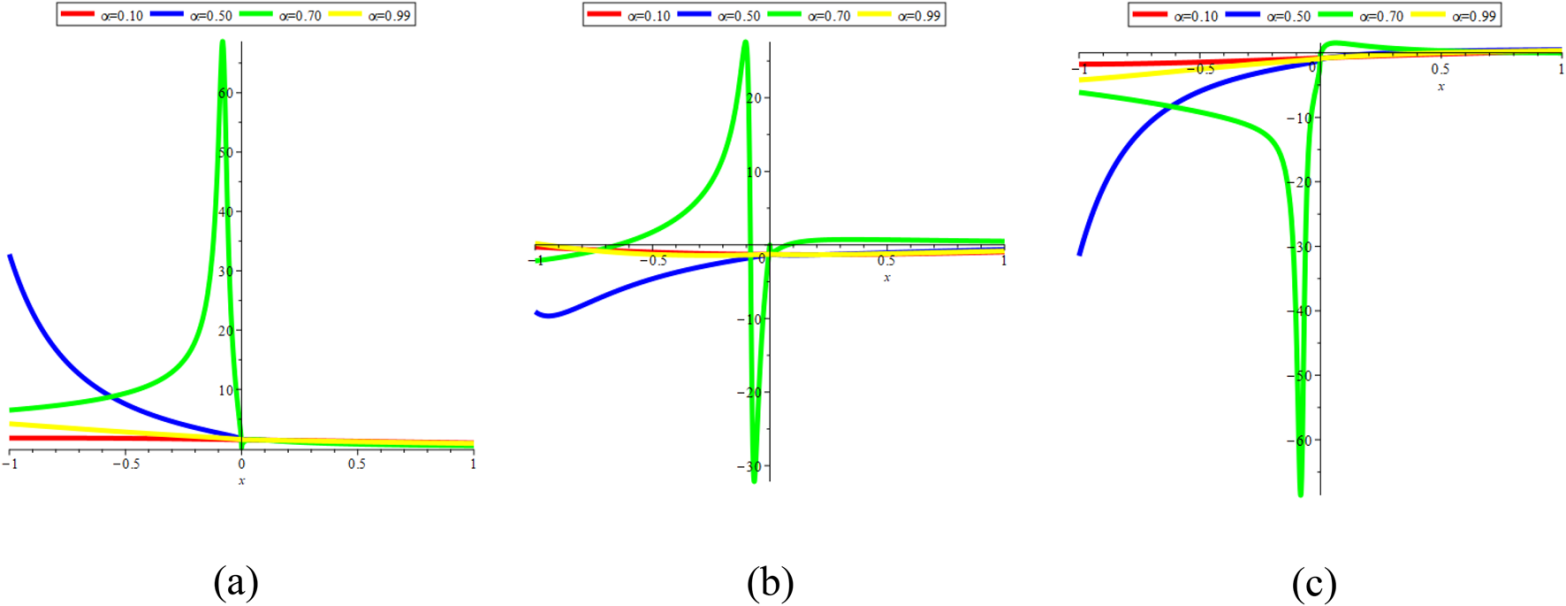
2D [(**a**) Absolute, (**b**) Real, (**c**) Complex] surfaces of $\psi_{1}^{ \pm } (x,t)$ for $\alpha = 0.10,\,\,0.50,\,\,0.70,\,\,0.99$ with $t = 0.01,$ $\beta = 0.25,$ $\mu = 1,$ $\lambda = 2,$ $k = 0.50,$ $\omega = 0.30,$ $\theta = 0.50,$ $a_{4} = 1,$ $a_{5} = 1,$ $a_{6} = 1,$ $- 30 \le x \le 30$.

In this sub-section, provide the effect of fractional parameter of the STFFL equation. In Fig. 19a–c, we present 2D [(a) Absolute, (b) Real, (c) Complex] surfaces of $\psi_{1}^{ \pm } (x,t)$ with different values of $\alpha = 0.10$ is represent the red line, $\alpha = 0.50$ is represent the blue line, $\alpha = 0.70$ is represent the green and $\alpha = 0.99$ is represent the yellow with $t = 0.01,$ $\beta = 0.25,$ $\mu = 1,$ $\lambda = 2,$ $k = 0.50,$ $\omega = 0.30,$ $\theta = 0.50,$ $a_{4} = 1,$ $a_{5} = 1,$ $a_{6} = 1,$ $- 30 \le x \le 30$.

**Figure 20:**
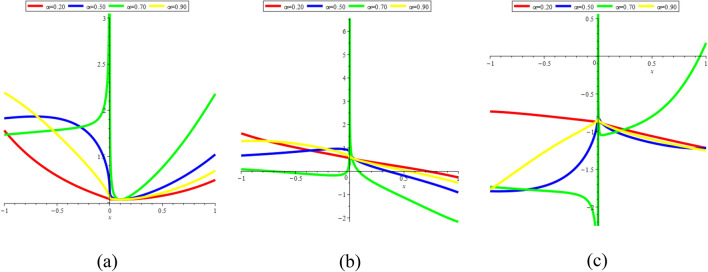
2D [(**a**) Absolute, (**b**) Real, (**c**) Complex] surfaces of $\psi_{3}^{ \pm } (x,t)$ for $\alpha = 0.20,\,\,0.50,\,\,0.70,\,\,0.90$ with $t = 0.01,$ $\beta = - 0.50,$ $\mu = 1,$ $\lambda = 2,$ $k = 0.50,$ $\omega = 0.30,$ $\theta = 0.50,$ $a_{4} = 1,$ $a_{5} = 1,$ $a_{6} = 1,$ $- 10 \le x \le 10$.

In Fig. 20a–c, we present 2D [(a) Absolute, (b) Real, (c) Complex] surfaces of $\psi_{3}^{ \pm } (x,t)$ with different values of $\alpha = 0.20$ is represent the red line, $\alpha = 0.50$ is represent the blue line, $\alpha = 0.70$ is represent the green and $\alpha = 0.90$ is represent the yellow with $t = 0.01,$ $\beta = - 0.50,$ $\mu = 1,$ $\lambda = 2,$ $k = 0.50,$ $\omega = 0.30,$ $\theta = 0.50,$ $a_{4} = 1,$ $a_{5} = 1,$ $a_{6} = 1,$ $- 10 \le x \le 10$.

**Figure 21:**
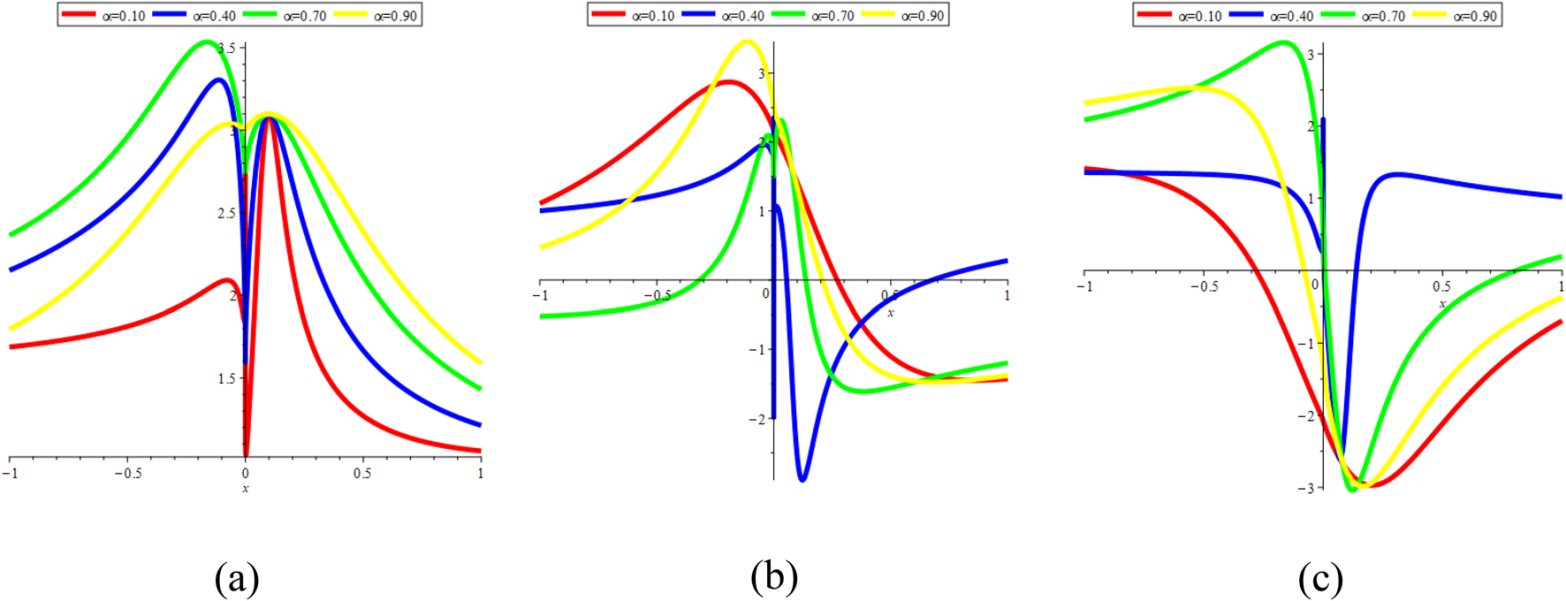
2D [(**a**) Absolute, (**b**) Real, (**c**) Complex] surfaces of $\psi_{4}^{ \pm } (x,t)$ for $\alpha = 0.10,\,\,0.40,\,\,0.70,\,\,0.90$ with $t = 0.01,$ $\beta = - 0.50,$ $\mu = 1,$ $\lambda = 2,$ $k = 0.50,$ $\omega = 0.30,$ $\theta = 0.50,$ $a_{4} = 1,$ $a_{5} = 1,$ $a_{6} = 1,$ $- 10 \le x \le 10$.

In Fig. 21a–c, we present 2D [(a) Absolute, (b) Real, (c) Complex] surfaces of $\psi_{4}^{ \pm } (x,t)$ with different values of $\alpha = 0.10$ is represent the red line, $\alpha = 0.40$ is represent the blue line, $\alpha = 0.70$ is represent the green and $\alpha = 0.90$ is represent the yellow with $t = 0.01,$ $\beta = - 0.50,$ $\mu = 1,$ $\lambda = 2,$ $k = 0.50,$ $\omega = 0.30,$ $\theta = 0.50,$ $a_{4} = 1,$ $a_{5} = 1,$ $a_{6} = 1,$ $- 10 \le x \le 10$.

**Figure 22:**
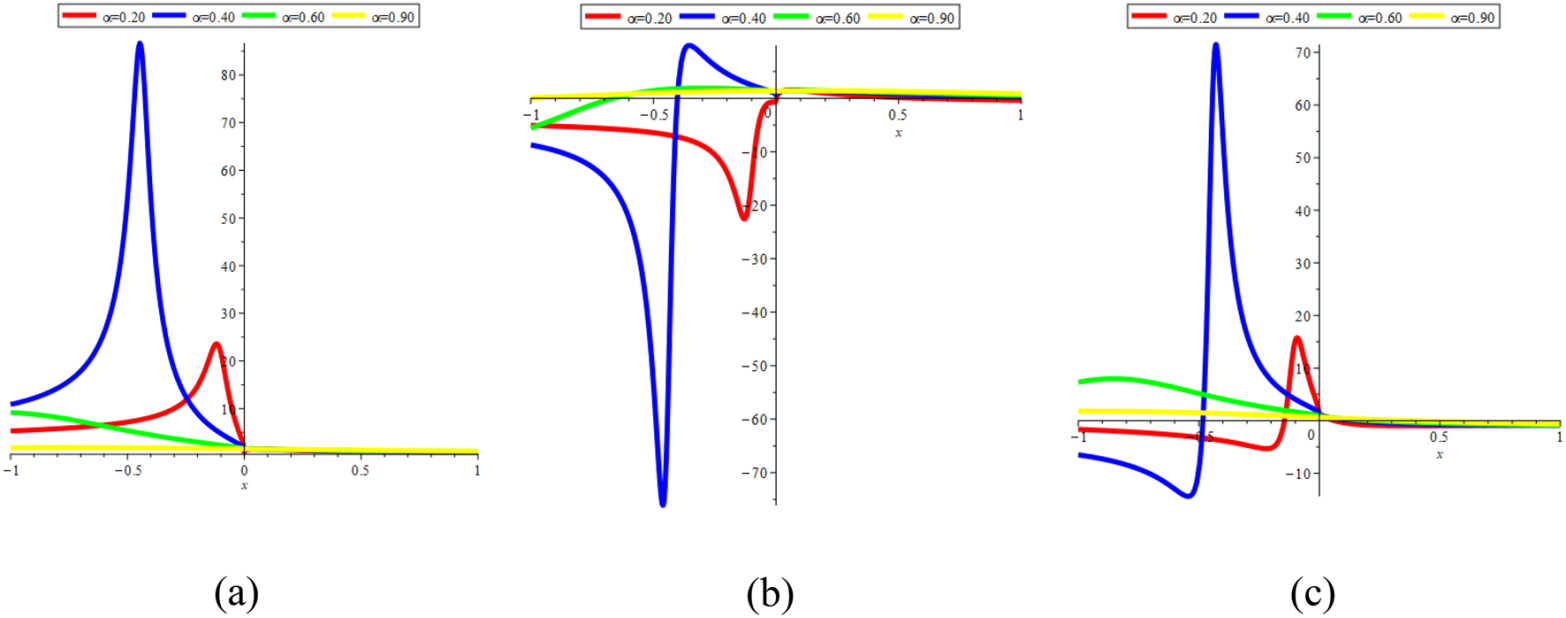
2D [(**a**) Absolute, (**b**) Real, (**c**) Complex] surfaces of $\psi_{7}^{ \pm } (x,t)$ for $\alpha = 0.20,\,\,0.40,\,\,0.60,\,\,0.90$ with $t = 0.01,$ $\beta = - 0.50,$ $\mu = 0.10,$ $\lambda = 0.20,$ $k = 0.50,$ $\omega = 0.30,$ $\theta = 0.50,$ $a_{4} = 1,$ $a_{5} = 1,$ $a_{6} = 1,$ $- 10 \le x \le 10$.

In Fig. 22a–c, we present 2D [(a) Absolute, (b) Real, (c) Complex] surfaces of $\psi_{7}^{ \pm } (x,t)$ with different values of $\alpha = 0.20$ is represent the red line, $\alpha = 0.40$ is represent the blue line, $\alpha = 0.60$ is represent the green and $\alpha = 0.90$ is represent the yellow with $t = 0.01,$ $\beta = - 0.50,$ $\mu = 0.10,$ $\lambda = 0.20,$ $k = 0.50,$ $\omega = 0.30,$ $\theta = 0.50,$ $a_{4} = 1,$ $a_{5} = 1,$ $a_{6} = 1,$ $- 10 \le x \le 10$.

**Figure 23:**
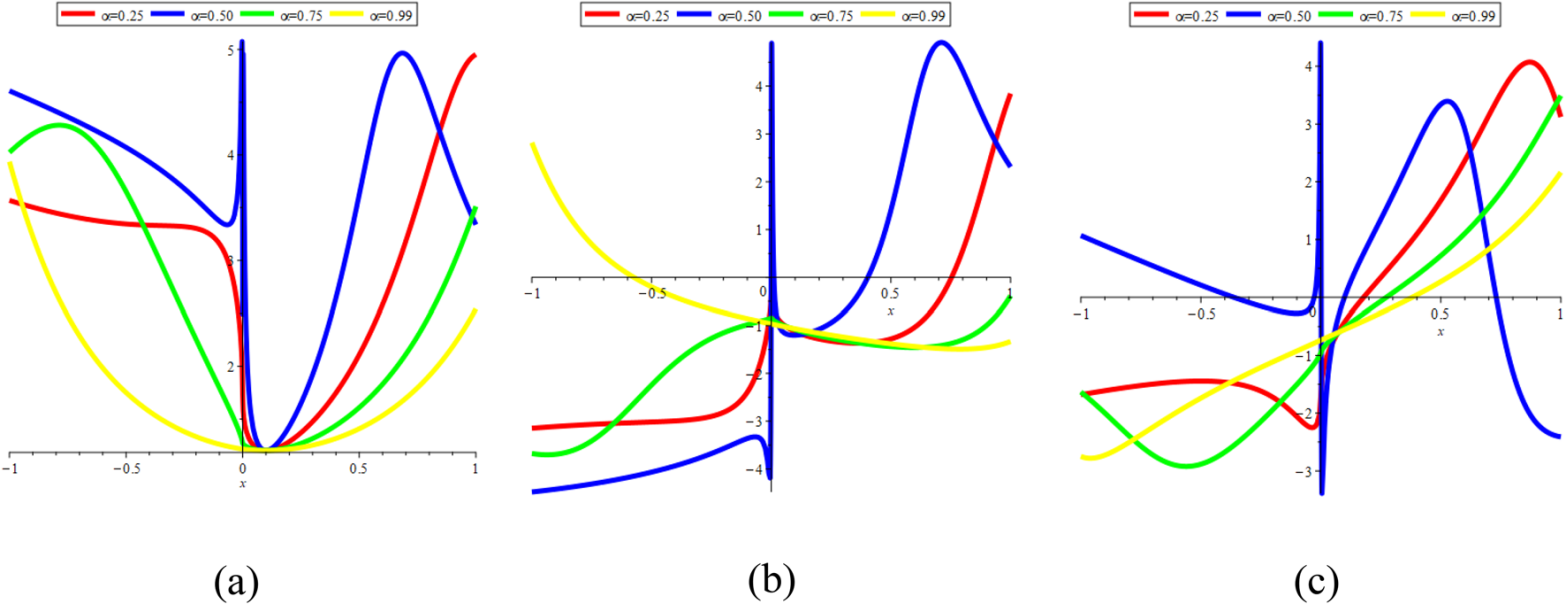
2D [(**a**) Absolute, (**b**) Real, (**c**) Complex] surfaces of $\psi_{12}^{ \pm } (x,t)$ for $\alpha = 0.25,\,\,0.50,\,\,0.75,\,\,0.99$ with $t = 0.01,$ $\beta = 0.50,$ $\mu = 1,$ $\lambda = 2,$ $k = 0.50,$ $\omega = 0.30,$ $\theta = 0.50,$ $a_{4} = 1,$ $a_{5} = 1,$ $a_{6} = 1,$ $- 10 \le x \le 10$.

In Fig. 23a–c, we present 2D [(a) Absolute, (b) Real, (c) Complex] surfaces of $\psi_{12}^{ \pm } (x,t)$ with different values of $\alpha = 0.25$ is represent the red line, $\alpha = 0.50$ is represent the blue line, $\alpha = 0.75$ is represent the green and $\alpha = 0.99$ is represent the yellow with $t = 0.01,$ $\beta = 0.50,$ $\mu = 1,$ $\lambda = 2,$ $k = 0.50,$ $\omega = 0.30,$ $\theta = 0.50,$ $a_{4} = 1,$ $a_{5} = 1,$ $a_{6} = 1,$ $- 10 \le x \le 10$.

## A comprehensive summary

The study delves into the space–time fractional Fokas–Lenells (STFFL) equation, an essential model in telecommunications and transmission technology that describes nonlinear pulse propagation in optical fibers. Employing the Sardar sub-equation (SSE) method within the STFFL framework, this research explores uncharted territories, unearthing a multitude of optical soliton solutions (OSSs) and conducting a comprehensive analysis of their bifurcations.

The discovered OSSs encompass a rich variety, spanning bright-dark, periodic, multiple bright-dark solitons, and other intriguing types, forming a captivating spectrum. These solutions unveil an intricate interplay among various soliton types, periodic sequences, breathers, coexisting solitons, as well as distinctive phenomena like kinks, anti-kinks, and dark-bell solitons. This exploration, rooted in an extensive literature review, uncovers previously undiscovered wave patterns within the dynamic STFFL equation, significantly expanding theoretical understanding and paving the way for innovative applications. Utilizing 2D, contour, and 3D diagrams, the study illustrates the influence of fractional and temporal parameters on these solutions. Moreover, a comprehensive analysis through various diagrams scrutinizes the nonlinear effects inherent in the STFFL equation. Employing a Hamiltonian function (HF) facilitates detailed phase-plane dynamics analysis, supplemented by simulations using Python and MAPLE software.

The practical implications of these discovered OSS solutions extend to real-world physical events, underscoring the effectiveness and applicability of the SSE scheme in solving time–space nonlinear fractional differential equations (TSNLFDEs). Acknowledging the SSE technique as a direct, efficient, and reliable numerical tool is crucial, illuminating precise outcomes in nonlinear comparisons.

The SSE technique successfully uncovers previously unexplored OSSs and their bifurcation analysis within the STFFL equation, encompassing a range of solutions showcased in visual representations. Investigating how wave profiles change for different derivative order and time parameters aids in understanding the nature and behavior of these OSSs.

The nonlinear effects of the STFFL equation are further explored through a variety of diagrams, establishing a Hamiltonian function that enhances the analysis of phase plane dynamics. These discovered OSS solutions hold significant implications for real-world physical events, endorsing the utility and reliability of the SSE scheme in elucidating various nonlinear fractional differential equations.

Ultimately, employing the SSE method for the STFFL equation showcases promising advantages in understanding diverse nonlinear phenomena across multiple fields, proving invaluable in unraveling intricate physical phenomena. The adaptable solutions with adjustable parameters underscore the method's remarkable effectiveness and adaptability in tackling complex nonlinear phenomena highlighted within this paper.

## Conclusion

We successfully applied the SSE technique to uncover unexplored OSSs and their bifurcation analysis (BA) within the STFFL equation. The derived OSSs include a range of solutions such as bright-dark, periodic, breather, multiple bright-dark solitons, kinks, anti-kinks, and dark-bell solitons, as depicted in Figs. 4, 5, 6, 7, 8. Additionally, contour surfaces were generated and presented in Figs. 9, 10, 11, 12, 13. Our examination explored how the wave profiles changed for different values of the derivative order and time parameters, illustrated in Figs. 14, 15, 16, 17, 18 and Figs. 19, 20, 21, 22, 23. Visual representations were crucial in understanding the nature and behavior of these OSSs. To investigate the nonlinear effects of the STFFL equation, we created 2D, 3D, contour, and BA diagrams. Establishing a Hamiltonian function (HF) furthered our analysis of the phase plane's (PP) dynamics. The discovered OSS solutions within the STFFL equation hold significant implications for real-world physical events. Our findings strongly support the utility and reliability of the SSE scheme for elucidating various nonlinear fractional differential equations. Ultimately, employing the SSE method for the STFFL equation unveils promising advantages in understanding diverse nonlinear phenomena spanning fields like nonlinear optics, quantum field theory, and solid-state physics. Providing adaptable solutions with adjustable parameters, this method proves invaluable in unraveling intricate nonlinear physical phenomena. The showcased solutions in this paper emphasize the method's remarkable effectiveness and adaptability in tackling these complex phenomena.

## Data Availability

Data will be available on request by contacting the corresponding author, Dr. Ahmed Refaie Ali, via ahmed.refaie@science.menofia.edu.eg, OR via Dr. Md. Nur Alam at nuralam23@pust.ac.bd.

## References

[1] Zhang H, Wang Y, Xu J (2020). Explicit monotone iterative sequences for positive solutions of a fractional differential system with coupled integral boundary conditions on a half-line. Adv. Differ. Equ..

[2] Raza N, Sial S, Kaplan M (2018). Exact periodic and explicit solutions of higher dimensional equations with fractional temporal evolution. Optik.

[3] Bazighifan O, Chatzarakis GE (2020). Oscillatory and asymptotic behavior of advanced differential equations. Adv. Differ. Equ..

[4] Awan AU, Rehman HU, Tahir M, Ramzan M (2020). Optical soliton solutions for resonant Schrödinger equation with anti-cubic nonlinearity. Optik.

[5] Rezazadeh H, Abazari R, Khater KM, Baleanu D (2020). New optical solitons of conformable resonant nonlinear Schrödinger’s equation. Open Phys..

[6] Arshed S, Raza N (2020). Optical solitons perturbation of Fokas–Lenells equation with full nonlinearity and dual dispersion. Chin. J. Phys..

[7] Raza N, Arshed S, Sial S (2019). Optical solitons for coupled Fokas–Lenells equation in birefringence fibers. Mod. Phys. Lett. B.

[8] Alam MN (2023). Soliton solutions to the electric signals in telegraph lines on the basis of the tunnel diode. Part. Differ. Equ. Appl. Math..

[9] Alam MN (2023). An analytical technique to obtain traveling wave solutions to nonlinear models of fractional order. Part. Differ. Equ. Appl. Math..

[10] Alam MN, Talib I, Tunç C (2023). The new soliton configurations of the 3D fractional model in arising shallow water waves. Int. J. Appl. Comput. Math..

[11] Ullah MS, Roshid HO, Ali MZ (2023). New wave behaviors of the Fokas–Lenells model using three integration techniques. PLoS ONE.

[12] Ahmad H, Alam MN, Rahman MA, Alotaibid MF, Omri M (2021). The unified technique for the nonlinear time-fractional model with the beta-derivative. Results Phys..

[13] Abdulazeez ST, Modanli M (2023). Analytic solution of fractional order Pseudo-Hyperbolic Telegraph equation using modified double Laplace transform method. Int. J. Math. Comput. Eng..

[14] Ismael HF, Baskonus HM, Bulut H, Gao W (2023). Instability modulation and novel optical soliton solutions to the Gerdjikov-Ivanov equation with M-fractional. Opt. Quant. Electron..

[15] Kumar S, Mohan B, Kumar R (2023). Newly formed center-controlled rouge wave and lump solutions of a generalized (3+1)-dimensional KdV-BBM equation via symbolic computation approach. Phys. Scr..

[16] Kumar S, Kumar A (2023). Newly generated optical wave solutions and dynamical behaviors of the highly nonlinear coupled Davey–Stewartson Fokas system in monomode optical fibers. Opt. Quant. Electron..

[17] Krishnan EV, Biswas A, Zhou Q, Alfiras M (2019). Optical soliton perturbation with Fokas–Lenells equation by mapping methods. Optik.

[18] Ullah MS, Seadawy AR, Ali MZ, Roshid HO (2023). Optical soliton solutions to the Fokas–Lenells model applying the φ6-model expansion approach. Opt. Quant. Electron..

[19] Ismael HF, Bulut H, Baskonus HM (2020). Optical soliton solutions to the Fokas–Lenells equation via sine-Gordon expansion method and (m+(G′/G))-expansion method. Pramana J. Phys..

[20] Elsherbeny AM, Mirzazadeh M, Akbulut A, Arnous AH (2023). Optical solitons of the perturbation Fokas Lenells equation by two different integration procedures. Optik.

[21] Chang L, Liu H, Xin X (2020). Lie symmetry analysis, bifurcations and exact solutions for the (2+1)-dimensional dissipative long wave system. J. Appl. Math. Comput..

[22] Houwe A, Abbagari S, Djorwe P, Saliou Y, Doka SY, Inc M (2022). W-shaped profile and breather-like soliton of the fractional nonlinear Schrödinger equation describing the polarization mode in optical fibers. Opt. Quant. Electron..

[23] Ma WX (2022). A novel kind of reduced integrable matrix mkdv equations and their binary darboux transformations. Mod. Phys. Lett. B.

[24] Faisal K, Abbagari S, Pashrashid A, Houwe A, Yao SW, Ahmad H (2023). Pure-cubic optical solitons to the Schrödinger equation with three forms of nonlinearities by Sardar subequation method. Results Phys..

[25] Raza N, Osman MS, Abdel-Aty AH, Abdel-Khalek S, Besbes HR (2020). Optical solitons of space-time fractional Fokas–Lenells equation with two versatile integration architectures. Adv. Differ. Equ..

[26] Abo-Seida OM, El-dabe NTM, Refaie Ali A, Shalaby GA (2021). Cherenkov FEL reaction with plasma-filled cylindrical waveguide in fractional D-dimensional space. IEEE Trans. Plasma Sci..

[27] Islam S, Halder B, Refaie Ali A (2023). Optical and rogue type soliton solutions of the (2+1) dimensional nonlinear Heisenberg ferromagnetic spin chains equation. Sci. Rep..

[28] Refaie Ali A, Eldabe NTM, El Naby AEHA (2023). EM wave propagation within plasma-filled rectangular waveguide using fractional space and LFD. Eur. Phys. J. Spec. Top..

[29] Yang XJ, Abdulrahman AA, Refaie Ali A (2023). An even entire function of order one is a special solution for a classical wave equation in one-dimensional space. Therm. Sci..

[30] Abdel-Aty A-H, Khater MMA, Attia RAM, Abdel-Aty M, Eleuch H (2020). On the new explicit solutions of the fractional nonlinear space-time nuclear model. Fractals.

[31] Osman MS, Tariq KU, Bekir A, Younis M, Abdel-Aty M (2020). Investigation of soliton solutions with different wave structures to the (2 + 1)-dimensional Heisenberg ferromagnetic spin chain equation. Commun. Theor. Phys..

[32] Hassan SM, Altwaty AA (2020). Optical solitons of the extended Gerdjikov–Ivanov equation in DWDM system by extended simplest equation method. Appl. Math. Inf. Sci..

[33] Abdel-Aty M, Furuichi S, Obada A-SF (2002). Entanglement degree of a nonlinear multiphoton Jaynes-Cummings model. J. Opt. B: Quant. Semiclass. Opt..

[34] Shapaan, M. DC conductivity, thermal stability and crystallization kinetics of the semiconducting 30P2O5 (50-x)V2O5 xB2O3 20Fe2O3 oxide glasses. *Int. J. Thin Film Sci. Technol.* **5**, 143–153 (2016).

[35] Jayamurugan, P., Ponnuswamy, V., Ashokan, S., & Mahalingam, T. Investigation on optical, morphological and thermal properties of spray coated polypyrrole film. Int. J. Thin Film Sci. Technol. **2**, 261–266 (2013).

[36] Mohamed HA, Hadia NMA (2015). Influence of post thermal annealing on the optical properties of SnO2 films prepared by electron beam evaporation technique. Int. J. Thin Film Sci. Technol..

[37] Thota S (2021). Implementation of a reducing algorithm for differential-algebraic systems in maple. Inf. Sci. Lett..

[38] Lorenz WE, Andres J, Franck G (2017). Fractal aesthetics in architecture. Appl. Math. Inf. Sci..

[39] Dinesh V, Murugesan G (2019). A CPW-fed hexagonal antenna with fractal elements for UWB applications. Appl. Math. Inf. Sci..

[40] Uthayakumar R, Gowrisankar A (2014). Generalized fractal dimensions in image thresholding technique. Inf. Sci. Lett..

[41] Mahmuda Maya MU, Alam MN, Refaie Ali A (2023). Influence of magnetic field on MHD mixed convection in lid-driven cavity with heated wavy bottom surface. Sci. Rep..

[42] Khan MH, Islam S, Refaie Ali A (2023). Certain results associated with lump and periodic-soliton solutions for (2+1)-D Calogero–Bogoyavlenskii–Schiff equation. J. Appl. Math. Stat. Anal..

[43] Justin M, David V, Shahen NHM (2022). Sundry optical solitons and modulational instability in Sasa-Satsuma model. Opt. Quant. Electron..

[44] Bashar MH, Islam S, Kumar D (2021). Construction of traveling wave solutions of the (2+1)-dimensional Heisenberg ferromagnetic spin chain equation. Part. Differ. Equ. Appl. Math..

[45] Shahen NHM, Foyjonnesa B, M. H., Tahseen, T., & Hossain, S. (2021). Solitary and rogue wave solutions to the conformable Time fractional modified Kawahara equation in mathematical physics. Adv. Math. Phys..

[46] Mamun AA, An T, Shahen NHM, Ananna SN, Foyjonnesa H, M. D., & Muazu, T. (2020). Exact and explicit travelling-wave solutions to the family of new 3D fractional WBBM equations in mathematical physics. Results Phys..

[47] Foyjonnesa S, N. H. M., & Rahman, M. M. (2022). Dispersive solitary wave structures with MI analysis to the unidirectional DGH equation via the unified method. Part. Differ. Equ. Appl. Math..

[48] Foyjonnesa S, N. H. M., Rahman, M. M., Alshomrani, A. S., & Inc, M. (2023). On fractional order computational solutions of low-pass electrical transmission line model with the sense of conformable derivative. Alex. Eng. J..

[49] Mamun, A. A., Ananna, S. N., An, T., Shahen, N. H. M., Asaduzzaman, M., & Foyjonnesa. (2021). Dynamical behaviour of travelling wave solutions to the conformable time-fractional modified Liouville and mRLW equations in water wave mechanics. *Heliyon* **7**(8), e07704 (2021). 10.1016/j.heliyon.2021.e07704 10.1016/j.heliyon.2021.e07704 PMC8350194 34401585

[50] Mamun, A. A., Shahen, N. H. M., Ananna, S. N., Asaduzzaman, M., & Foyjonnesa. Solitary and periodic wave solutions to the family of new 3D fractional WBBM equations in mathematical physics. *Heliyon* **7**(7), e07483 (2021). 10.1016/j.heliyon.2021.e07483 10.1016/j.heliyon.2021.e07483 PMC8273412 34286141

[51] Ghanbari, B., & Gómez‐Aguilar, J. Optical soliton solutions for the nonlinear Radhakrishnan–Kundu–Lakshmanan equation. Mod. Phys. Lett. B **33**(32), 1950402 (2019). 10.1142/s0217984919504025

[52] Ghanbari B, Gómez-Aguilar J (2019). New exact optical soliton solutions for nonlinear Schrödinger equation with second-order spatio-temporal dispersion involving M-derivative. Mod. Phys. Lett. B.

[53] Ghanbari B, Băleanu D (2020). New optical solutions of the fractional Gerdjikov-Ivanov equation with conformable derivative. Front. Phys..

[54] Khater MMA, Ghanbari B (2021). On the solitary wave solutions and physical characterization of gas diffusion in a homogeneous medium via some efficient techniques. Eur. Phys. J. Plus.

[55] Ghanbari B (2019). Abundant soliton solutions for the Hirota–Maccari equation via the generalized exponential rational function method. Mod. Phys. Lett. B.

[56] Ghanbari B, Kuo C (2019). New exact wave solutions of the variable-coefficient (1 + 1)-dimensional Benjamin-Bona-Mahony and (2 + 1)-dimensional asymmetric Nizhnik-Novikov-Veselov equations via the generalized exponential rational function method. The European Physical Journal Plus.

[57] Alam MN, Rahim MA, Hossain MN, Tunç C (2023). Dynamics of damped and undamped wave natures of the fractional Kraenkel–Manna–Merle system in ferromagnetic materials. J. Appl. Comput. Mech..

[58] Alam MN, Akash HS, Saha U, Hasan MS, Parven MW, Tunç C (2023). Bifurcation analysis and solitary wave analysis of the nonlinear fractional soliton neuron model. Iran. J. Sci..

[59] Alam MN, Islam SMR (2023). The agreement between novel exact and numerical solutions of the nonlinear models. Part. Differ. Equ. Appl. Math..

